# Effects of acupuncture on cognitive function and lipid metabolism in post-stroke vascular dementia: a systematic review and meta-analysis of randomized controlled trials

**DOI:** 10.3389/fnagi.2026.1797567

**Published:** 2026-06-17

**Authors:** Mengqi Li, Tao Liu, Wei Zhao, Jingxi Zhao, Duo Zhao, Xuezhu Zhang

**Affiliations:** 1 National Clinical Research Center for Chinese Medicine, Tianjin, China; 2 First Teaching Hospital of Tianjin University of Traditional Chinese Medicine, Tianjin, China; 3 Tianjin University of Traditional Chinese Medicine, Tianjin, China; 4 Tianjin Normal University, Tianjin, China; 5 Xi’an Jiaotong Liverpool University, Suzhou, Jiangsu, China

**Keywords:** acupuncture, cognitive function, meta-analysis, safety, stroke, vascular dementia

## Abstract

**Introduction:**

Post-stroke vascular dementia (PSVD) is a prevalent cognitive disorder and an underexplored area in dementia research, yet conventional pharmacotherapies have limited efficacy. Although acupuncture has emerged as a promising non-pharmacological intervention, no systematic review has specifically evaluated its effects in PSVD. This meta-analysis therefore aimed to assess the efficacy, safety, and potential treatment parameters of acupuncture for PSVD.

**Methods:**

Following the Preferred Reporting Items for Systematic Reviews and Meta-Analyses (PRISMA) guidelines, seven databases were searched for randomized controlled trials (RCTs) investigating acupuncture for PSVD from inception to November 3, 2025. Included studies were assessed for risk of bias using the Cochrane Risk-of-Bias 2.0 tool, and meta-analyses were conducted in Stata to evaluate outcomes and explore sources of heterogeneity. The main pooled analyses included both add-on trials and monotherapy trials, which addressed distinct clinical questions and were therefore interpreted cautiously.

**Results:**

Thirteen RCTs involving 1,085 patients were included. Acupuncture was associated with a higher study-defined overall effective rate, a non-standardized and heterogeneous outcome [relative risk = 1.27, 95% confidence interval (CI): 1.17–1.37, *p* < 0.001], as well as improvements in Mini-Mental State Examination (MMSE) scores [mean difference (MD) = 2.89, 95% CI: 2.15–3.64, *p* < 0.001] and Montreal Cognitive Assessment (MoCA) scores (MD = 2.80, 95% CI: 2.33–3.28, *p* < 0.001). An exploratory subgroup analysis suggested a larger MMSE effect in trials with 12 weeks of treatment (MD = 4.20). However, because this analysis was not prespecified and the number of studies in each subgroup was limited, this finding should be interpreted as hypothesis-generating. Acupuncture was also associated with lower total cholesterol [standardized mean difference (SMD) = –0.574, *p* = 0.002] and triglyceride levels (SMD = –0.552, *p* = 0.002), although these lipid-related findings were based on only two studies and should be regarded as exploratory. Only three minor adverse events (scalp hematomas) were reported; however, adverse-event reporting was incomplete across the included studies. No strong signal of publication bias was detected for MMSE.

**Discussion:**

Overall, acupuncture-related interventions may be associated with cognitive improvement in PSVD, but the certainty of evidence remains limited by methodological weaknesses, incomplete safety reporting, and the exploratory nature of the study-defined overall effective rate, lipid-related outcomes, and treatment-duration findings. Larger, high-quality multicenter RCTs are needed to confirm these observations.

**Systematic review registration:**

https://www.crd.york.ac.uk/prospero/display_record.php?ID=CRD420251246224, identifier CRD420251246224.

## 1 Introduction

Post-stroke vascular dementia (PSVD) is a distinct cognitive disorder that results from cerebrovascular damage after an acute stroke (Filler et al., 2023; Chung et al., 2024). PSVD represents a significant global public health burden (Khan et al., 2025). According to the most recent data, updated in 2021, stroke is the third leading cause of death (7.3 million deaths, or 10.7% of all deaths globally) and the fourth leading contributor to disability-adjusted life years (DALYs) lost (160.5 million) (Feigin et al., 2024a,b). Stroke is the leading cause of death in China, leading to permanent disability in 12.5% of survivors (Tu et al., 2023a,b). In addition to paralysis, stroke may also cause severe brain dysfunction; approximately half of stroke survivors experience substantial brain dysfunction (Lo et al., 2019; Pendlebury and Rothwell, 2019), and 20–30% of them go on to develop dementia during the first year (Rost et al., 2022). Prospective cohort studies have indicated that the probability of developing dementia after intracerebral hemorrhage (ICH) rises to 19.0% 6 months after ICH, 14.2% 1 year after ICH, 19.8% 2 years after ICH, 24.5% 3 years after ICH, 28.3%–35.0% 4 years after ICH, and 32.0%–37.4% 5 years after ICH (Jin et al., 2025). Compared with the general population, stroke survivors have a substantially increased risk of dementia, with recurrent stroke further increasing this risk. Recurrent vascular events may further aggravate both functional disability and cognitive decline, creating a reinforcing cycle between the two conditions (Joundi et al., 2025). Vascular dementia accounts for approximately 15% of all dementia cases, and PSVD is a clinically important subtype within the spectrum of vascular cognitive impairment after stroke (O’Brien and Thomas, 2015). It refers to cognitive dysfunction beginning 3–6 months post-radiologically verified stroke (Rost et al., 2022; Filler et al., 2023).

Rather than following the typical pathological pattern of Alzheimer’s disease, PSVD is mainly related to vascular lesions affecting cognition-relevant regions, such as the thalamus and angular gyrus, as well as disruption of fronto-subcortical pathways. In addition to white matter degeneration, PSVD is also associated with synaptic dysfunction and neuroinflammation (Zlokovic, 2011; Sun et al., 2014; Kim et al., 2022). Although vascular and neurodegenerative disorders are strongly linked, effective prevention and treatment remain challenging (Farooq et al., 2017). Available pharmacological treatments, including acetylcholinesterase inhibitors, N-methyl-D-aspartate (NMDA) inhibitors (e.g., memantine), and calcium channel blockers (e.g., nimodipine) (Farooq et al., 2017; Battle et al., 2021) have limited efficacy and are often associated with adverse effects, such as gastrointestinal problems, hypotension, dizziness, and psychiatric symptoms (Sun, 2018).

As a form of Traditional Chinese medicine, acupuncture is a treatment modality that has been gaining popularity as a complementary therapy in the past 2,000 years (Han et al., 2021). Proposed mechanisms include reductions in oxidative stress and neuroinflammation, improvement of cerebral perfusion and synaptic plasticity, regulation of glucose metabolism and neurotransmission, and optimization of cerebral blood flow (Ma et al., 2020; Li et al., 2022). Acupuncture is generally considered to have fewer reported adverse effects than pharmacotherapy when administered properly (Witt et al., 2005). For patients with poor adherence to rehabilitation, it may also represent a practical treatment option. Clinical evidence suggests that acupuncture can have synergistic effects with medications that improve cerebral circulation or cognitive training (Su et al., 2021). However, current randomized controlled trials (RCTs) have reported inconsistent findings, and some studies have shown limited benefit (Wen et al., 2022).

These inconsistencies point to a limited and still incomplete evidence base. Although systematic reviews have examined acupuncture for vascular dementia in general (Ng et al., 2025), none has specifically focused on PSVD, which has distinct pathophysiology and a different clinical time course. With vascular dementia becoming increasingly common in aging populations with greater vascular risk exposure, a focused evaluation of PSVD is warranted. Therefore, this systematic review and meta-analysis synthesized randomized evidence on acupuncture-related interventions for PSVD, focusing on cognitive outcomes, reported safety, methodological quality assessed with the Cochrane Risk of Bias 2.0 tool, and treatment-related factors such as frequency and duration. Addressing these objectives will inform the development of evidence-based guidelines for incorporating acupuncture into comprehensive PSVD management strategies.

## 2 Methods

### 2.1 Study design

The study followed the Preferred Reporting Items for Systematic Reviews and Meta-Analyses (PRISMA) guidelines (Page et al., 2021), which were last modified in 2020. Additionally, we submitted this review’s protocol online to the International Prospective Register of Systematic Reviews (PROSPERO, CRD420251246224).

### 2.2 Literature search strategy

A thorough search of the literature was conducted using a variety of databases, including PubMed, Embase, the Cochrane Library, Web of Science, CNKI, VIP, and WanFang, from the database’s inception to November 3, 2025. Two authors, Mengqi Li and Tao Liu, conducted the database searches. The search terms included “acupuncture,” “electroacupuncture,” “stroke,” “vascular dementia,” etc. The complete retrieval strings for each of the seven databases are presented in Supplementary Table 1.

### 2.3 Inclusion and exclusion criteria

Inclusion criteria: (1) adults aged ≥ 50 years with clinically and radiologically confirmed post-stroke vascular dementia (PSVD), meeting standardized diagnostic criteria such as NINDS-AIREN, DSM-5, or ICD-11; (2) Intervention: acupuncture-based therapies (manual acupuncture, electroacupuncture, or scalp acupuncture) as the primary intervention; (3) Control: any comparator excluding acupuncture (e.g., sham acupuncture, pharmacological agents like donepezil or nimodipine, or placebo); (4) Outcomes: Mini-Mental State Examination (MMSE), Montreal Cognitive Assessment (MoCA), total cholesterol (TC), triglycerides (TG), low-density lipoprotein cholesterol (LDL-C); (5) Study type: RCTs.

Exclusion criteria: (1) studies without random assignment; (2) studies with insufficient data for quantitative synthesis, defined as missing essential numerical information such as sample sizes, means, standard deviations, change scores, event counts, or response-category counts, when these data could not be derived from the text, tables, or figures or obtained from the authors; (3) studies lacking control groups; (4) studies with apparent data flaws that prevented reliable data extraction or analysis, such as inconsistent sample sizes across the text and tables, mismatched denominators, percentages inconsistent with event counts, impossible or implausible numerical values, or unresolved duplicate or overlapping data; (5) duplicated studies; (6) interventions not primarily acupuncture-based (e.g., acupressure only, laser acupuncture); (7) non-PSVD populations (e.g., Alzheimer’s disease, traumatic brain injury); (8) animal tests; and (9) retracted articles.

### 2.4 Definition of study-defined overall effective rate

In this review, overall effective rate was extracted as a study-defined composite response outcome rather than as a universally standardized or validated endpoint. In the included trials, participants were usually classified into response categories such as “markedly effective,” “effective,” “improved,” or “ineffective,” according to the criteria specified by the original investigators. The study-defined overall effective rate was calculated using the numerator definition reported in each original study, most commonly as the number of participants classified as “markedly effective” plus “effective” divided by the total number of participants, although some trials included additional improvement categories in the numerator. Because the response categories and calculation criteria varied across studies, this outcome was treated as a non-standardized exploratory outcome. The study-level definitions and calculation methods are summarized in Supplementary Table 2.

### 2.5 Data extraction

Two individual researchers, Mengqi Li and Tao Liu, summarized the results: first author, year of publication, origin of study, study design, number of participants, age of participants, intervention, treatment duration, control group, and outcome measures (Table 1). Disagreements were resolved by consultation with a third researcher, Wei Zhao.

**TABLE 1:** Characteristics of the included studies.

References	Sample size		Number of cases (M/F)		Age (years)		Course (months)		Tre ament		Course of treatment (weeks)	Outcomes
	T	C	T	C	T	C	T	C	T	C		
Li et al. (2025)	31	31	16/15	16/15	58 ± 7	57 ± 6	23.87 ± 9.90	25.03 ± 9.66	Acupuncture + Active control	Standard care + Oral Donepezil	8	① ② ③ ④
Qiao and Hu (2023)	40	40	25/15	25/15	69.13 ± 6.31	68.21 ± 6.11	24.12 ± 3.72	25.32 ± 5.04	Acupuncture + Active control +	Clopidogrel Bisulfate Butylphthalide soft capsules	3	⑤ ⑥ ⑦
Han et al. (2021)	59	59	30/29	29/30	64 ± 9	66 ± 8	6.3 ± 2.6	6.7 ± 24	Acupuncture + Active control	Oxiracetam capsules	8	① ② ④
Tong (2020)	60	60	39/21	40/20	63.2 ± 8.9	63.4 ± 9.7	NA	NA	Acupuncture + Rehabilitation	Conventional medication + Rehabilitation	12	②
Feng and Wang (2020)	30	30	17/13	16/14	65.77 ± 5.25	64.30 ± 5.98	14.27 ± 2.52	14.73 ± 1.74	Acupuncture + Active control	Conventional medication + Rehabilitation	8	① ②
Cheng (2020)	30	30	16/14	17/13	62.22 ± 8.64	62.19 ± 8.79	2.11 ± 0.83	2.06 ± 0.71	Acupuncture + Active control	Xingnaojing injection	6	② ③
Zheng (2016)	45	45	30/15	28/17	64.35 ± 6.72	66.48 ± 6.84	1.11 ± 0.21	1.11 ± 0.21	Acupuncture + Active control	Conventional medication + Rehabilitation	12	① ② ④
Kong (2013)	64	64	43/21	41/23	65.27 ± 5.12	66.73 ± 5.51	11.07 ± 4.08	10.85 ± 3.96	Acupuncture + Active control	Standard care + Oral Piracetam	4	① ②
Gao et al. (2013)	30	30	13/17	14/16	71.57 ± 5.04	72.27 ± 4.08	5.63 ± 2.08	5.00 ± 1.85	Acupuncture + Active control	Standard care	8	② ③
Liu et al. (2008)	47	45	30/17	29/16	65 ± 1	66 ± 1	NA	NA	Acupuncture monotherapy	Nimodipine tablets	4	① ②
Lun et al. (2004a)	57	32	35/22	20/12	60.52 ± 7.30	58.85 ± 6.83	NA	NA	Acupuncture monotherapy	Oral nimotop	9	①
Lun et al. (2004b)	25	25	NA	NA	NA	NA	NA	NA	Acupuncture monotherapy	Oral nimotop	9	①
Liu (2003)	46	30	25/21	16/14	64.31 ± 10.27	63.19 ± 9.45	NA	NA	Acupuncture monotherapy	Dextran 40 + compound Danshen injection	8	⑤ ⑥ ⑦

M/F, Male/Female; NA, Data were not available in the primary study. ① Study-defined overall effective rate; ② Mini-Mental State Examination (MMSE); ③ Montreal Cognitive Assessment (MoCA); ④ total cholesterol (TC); ⑤ triglycerides (TG); ⑥ low-density lipoprotein cholesterol (LDL-C).

### 2.6 Quality assessment

We evaluated the quality of the RCTs using the Cochrane Risk of Bias Tool (RoB 2) (Sterne et al., 2019). This tool assesses five core bias domains: bias arising from the randomization process, bias due to intervention deviations, bias due to missing outcome data, bias in outcome measurement, and bias in the selection of the reported result. Two reviewers (Mengqi Li and Tao Liu) independently rated each domain as low risk, some concerns, or high risk of bias according to RoB 2 criteria. Discrepancies were resolved through discussion and consultation with a third independent reviewer (Wei Zhao). The domain-specific judgments were synthesized to determine the overall risk of bias for each trial.

### 2.7 Certainty of evidence

A formal GRADE assessment was not originally planned or undertaken as a full Summary of Findings rating exercise in this review. However, to improve the transparency and interpretability of the findings, we added a GRADE-informed narrative appraisal of the certainty of evidence for the main outcomes. This appraisal considered the major domains commonly used in GRADE, including risk of bias, inconsistency, indirectness, imprecision, and potential publication bias. The certainty of evidence was interpreted cautiously when the evidence base was affected by unclear or high risk of bias, substantial heterogeneity, small numbers of studies or participants, non-standardized outcome definitions, incomplete adverse-event reporting, or limited ability to assess publication bias. The GRADE-informed narrative appraisal is summarized in Supplementary Table 4.

### 2.8 Statistical analysis

All statistical analyses were performed using Stata 17 software (Stata, 2022). The mean difference (MD) was used for quantitatively evaluating data using the same scale, whereas the standardized mean difference (SMD) was used for outcomes measured on different scales. Risk ratios (RRs) were used for dichotomous outcomes. Effect sizes were reported with 95% confidence intervals (CIs). Heterogeneity was tested for each outcome. Model selection was informed by both statistical heterogeneity and clinical or methodological considerations, including differences in acupuncture regimens, comparator interventions, intervention design, treatment duration, and outcome definitions. To examine whether the findings depended on model choice, sensitivity analyses comparing fixed-effect and random-effects models were conducted for the main outcomes where feasible.

Subgroup analyses were conducted where data permitted. Subgroup analysis by intervention design was planned based on clinical relevance, because add-on trials and monotherapy trials address distinct clinical questions. Add-on trials were defined as studies comparing acupuncture combined with conventional therapy versus conventional therapy alone, addressing whether acupuncture provided additional benefit beyond conventional care. Monotherapy trials were defined as studies comparing acupuncture alone with a non-acupuncture comparator, addressing the relative effect of acupuncture as the principal intervention. Because these designs answer distinct clinical questions, pooled estimates combining them were interpreted cautiously. Subgroup analysis by treatment duration was conducted *post-hoc* to explore potential sources of heterogeneity and was interpreted as exploratory and hypothesis-generating. Funnel plots, the Egger test (Egger et al., 1997), and the Begg test (Begg and Mazumdar, 1994) were used to assess publication bias when at least ten studies were available. All statistical tests were two-sided, and *p* < 0.05 was considered statistically significant. The official Cochrane RoB 2.0 online assessment tool (Sterne et al., 2019) was used to complete the risk of bias assessment and generate the risk of bias summary graph and summary figure for all included studies.

## 3 Results

### 3.1 Study selection

**FIGURE 1:**
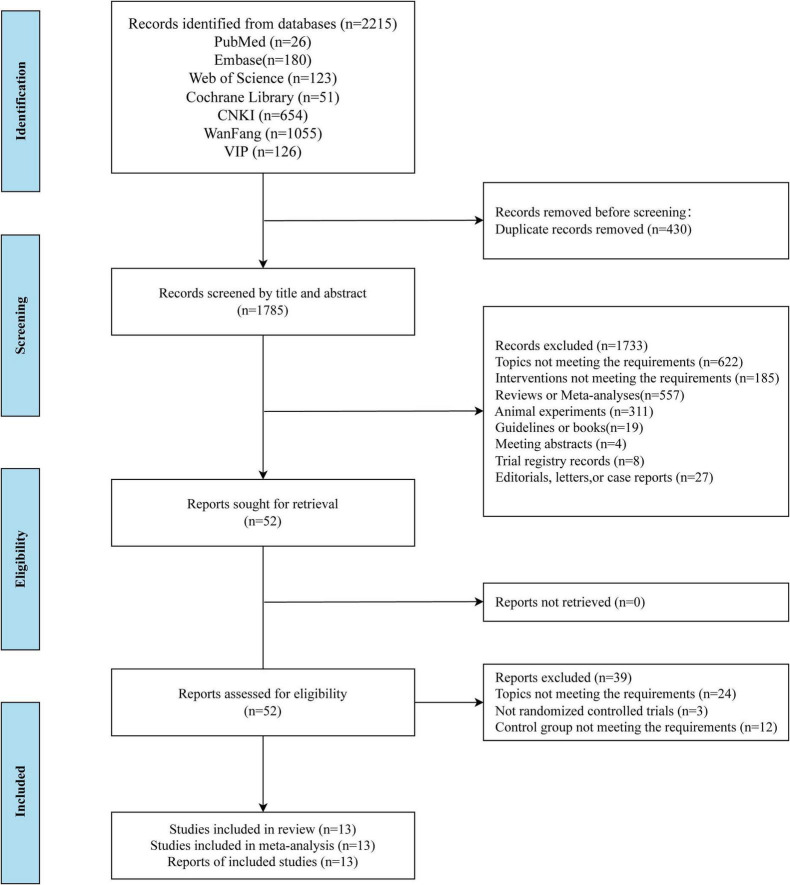
PRISMA flow diagram for study selection.

This study was carried out in accordance with the PRISMA guidelines, and the study selection process is shown in Figure 1. A total of 2,215 records were identified from seven databases. After duplicate records were removed, 1,785 records were screened by title and abstract, of which 1,733 were excluded. Fifty-two reports were sought for retrieval and assessed for eligibility. Thirty-nine reports were excluded with reasons, resulting in 13 studies, represented by 13 reports, being included in the review and meta-analysis (Zheng, 2016; Tong, 2020; Qiao and Hu, 2023; Lun et al., 2004b,2004a; Liu et al., 2008; Liu, 2003; Li et al., 2025; Kong, 2013; Han et al., 2021; Gao et al., 2013; Feng and Wang, 2020; Cheng, 2020).

### 3.2 Study characteristics

As shown in Table 1, 13 studies involving 1,085 patients were included, with 564 patients in the treatment group and 521 in the control group. Sample sizes ranged from 25 to 64. The publication years of the literature ranged from 2003 to 2025. All 13 included studies were conducted in China and published in Chinese. Only three studies (Liu et al., 2008; Feng and Wang, 2020; Li et al., 2025) reported adverse events; the remaining studies did not mention them. Furthermore, no study identified a statistically significant difference in baseline information.

### 3.3 Risk of bias

**FIGURE 2:**
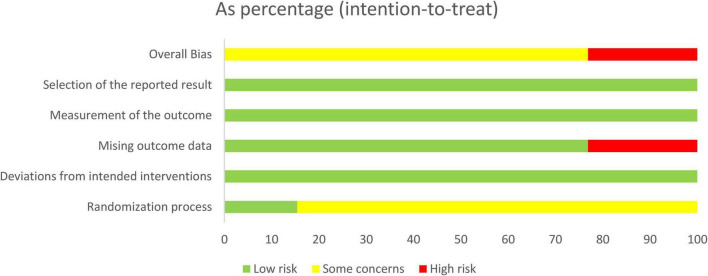
Risk-of-bias summary of the included studies assessed using the Cochrane RoB 2.0 tool.

**FIGURE 3:**
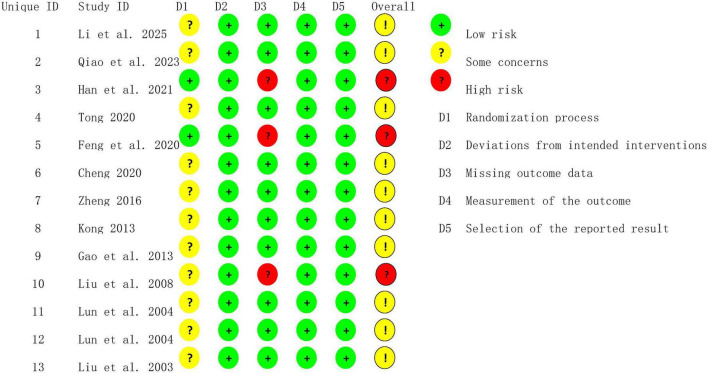
Risk-of-bias traffic light plot for the included studies assessed using the Cochrane RoB 2.0 tool.

The RoB 2 assessment results for the 13 included RCTs, summarized in Figures 2, 3, showed that no study was rated as overall low risk of bias: 80% (10/13) had some concerns and 20% (3/13) had high risk. Specifically, 15% (2/13) of studies were at low risk in the randomization process, whereas 85% (11/13) had some concerns because allocation concealment was insufficiently reported; no study was rated as high risk in this domain. All 13 studies were at low risk for deviations from intended interventions, measurement of the outcome, and selection of the reported result. In addition, 80% (10/13) were at low risk for missing outcome data, whereas 20% (3/13) were at high risk because the handling of missing data was unclear. These limitations in randomization and missing outcome data were the main sources of potential bias and should be considered when interpreting the results. Publication bias was assessed using funnel plots when a meta-analysis included 10 or more studies. Additionally, a leave-one-out sensitivity analysis was performed, whereby one study was systematically removed and the data were reanalyzed to determine the effect of each study on the overall effect estimate.

#### 3.3.1 Overall effective rate

**FIGURE 4:**
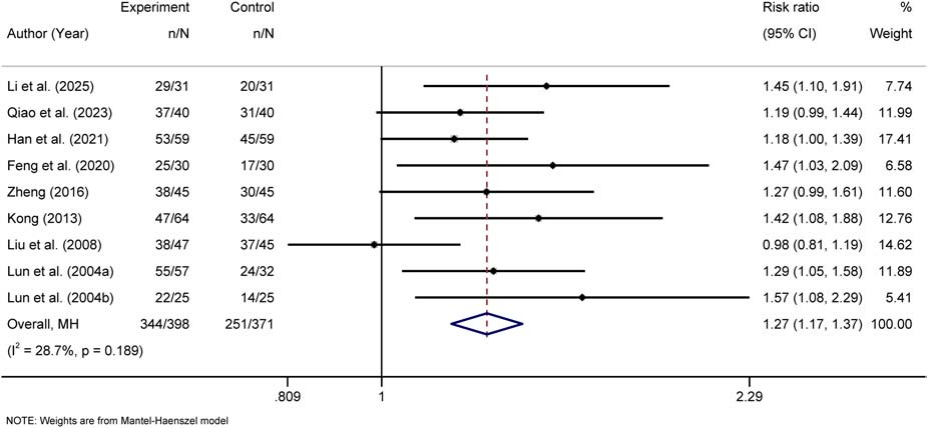
Forest plot showing the effect of the experimental and control groups on the efficacy rate of PSVD.

**FIGURE 5:**
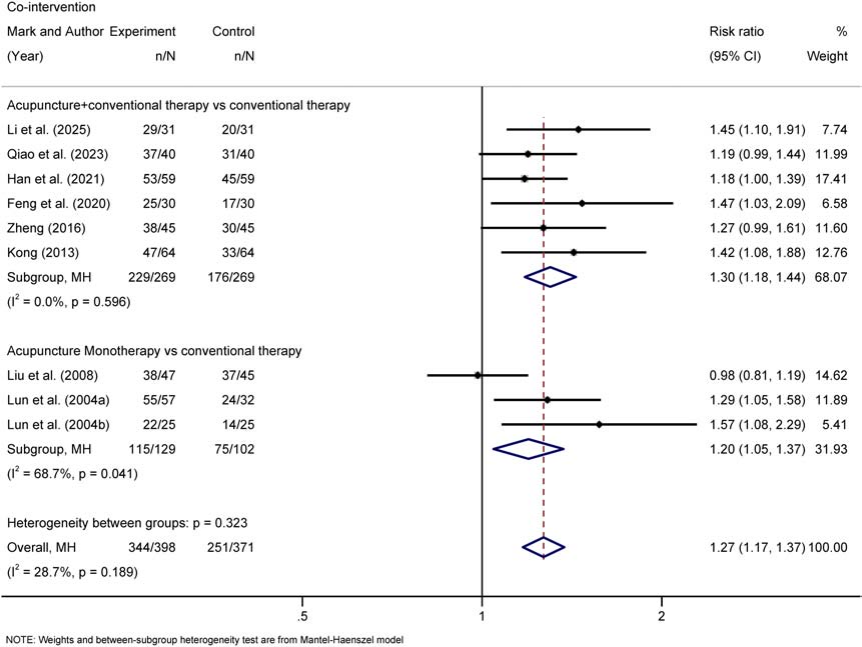
Forest plot of subgroup analysis on efficacy rates in PSVD.

A total of nine studies (Lun et al., 2004a,2004b; Liu et al., 2008; Kong, 2013; Zheng, 2016; Feng and Wang, 2020; Han et al., 2021; Qiao and Hu, 2023; Li et al., 2025) involving 769 patients were considered. The results of the analysis are shown in Figure 4. This outcome was not based on a universally standardized or validated endpoint; rather, it reflected the response categories and calculation criteria used in the original trials, as summarized in Supplementary Table 2. In the primary analysis, a fixed-effect model was used; sensitivity analyses comparing fixed-effect and random-effects models are summarized in Supplementary Table 5. Across trials evaluating acupuncture either as an add-on to conventional therapy or as a monotherapy comparator, acupuncture-based interventions were associated with a higher study-defined overall effective rate than control interventions (RR = 1.27, 95% CI = 1.17–1.37, *p* < 0.001). However, this finding should be interpreted as exploratory and supportive only, because the definitions and calculation criteria for response varied across studies. Subgroup analyses by intervention design (Figure 5) showed that add-on acupuncture combined with conventional therapy (six trials, *n* = 538) (Kong, 2013; Zheng, 2016; Feng and Wang, 2020; Han et al., 2021; Qiao and Hu, 2023; Li et al., 2025) was associated with a higher study-defined overall effective rate than conventional therapy alone (RR = 1.30, 95% CI = 1.18–1.44, *p* < 0.001), with no heterogeneity (*I*^2^ = 0, *p* = 0.596). This subgroup addresses whether acupuncture provides additional benefit when added to conventional therapy. Among the three trials evaluating acupuncture monotherapy (*n* = 231) (Lun et al., 2004a,2004b; Liu et al., 2008), the study-defined overall effective rate favored acupuncture over non-acupuncture comparators (RR = 1.20, 95% CI = 1.05–1.37, *p* = 0.009), with moderate between-study heterogeneity (*I*^2^ = 68.7%, *p* = 0.041). This subgroup addresses the relative effect of acupuncture as the principal intervention rather than its additive effect. Because the number of monotherapy trials was small, this result should be interpreted cautiously. The point estimate of combined therapy was found to be 8.4% higher in meta-regression analysis (β = 0.084, *p* = 0.433). Nonetheless, the difference was not statistically significant.

#### 3.3.2 MMSE

**FIGURE 6:**
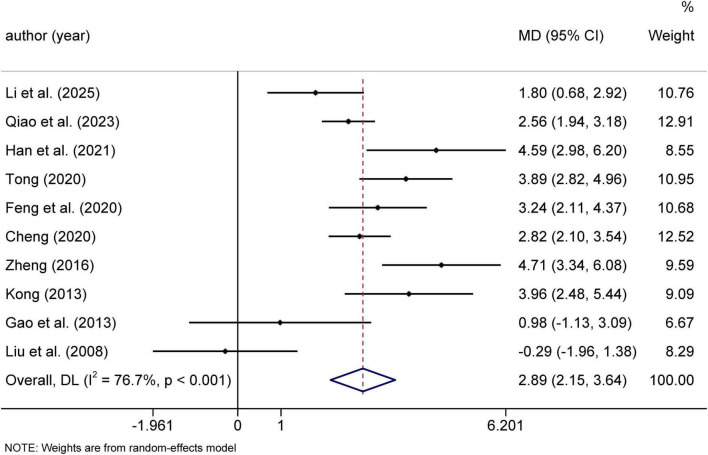
Forest plot of the effect of the experimental group and control group on MMSE of PSVD.

**FIGURE 7:**
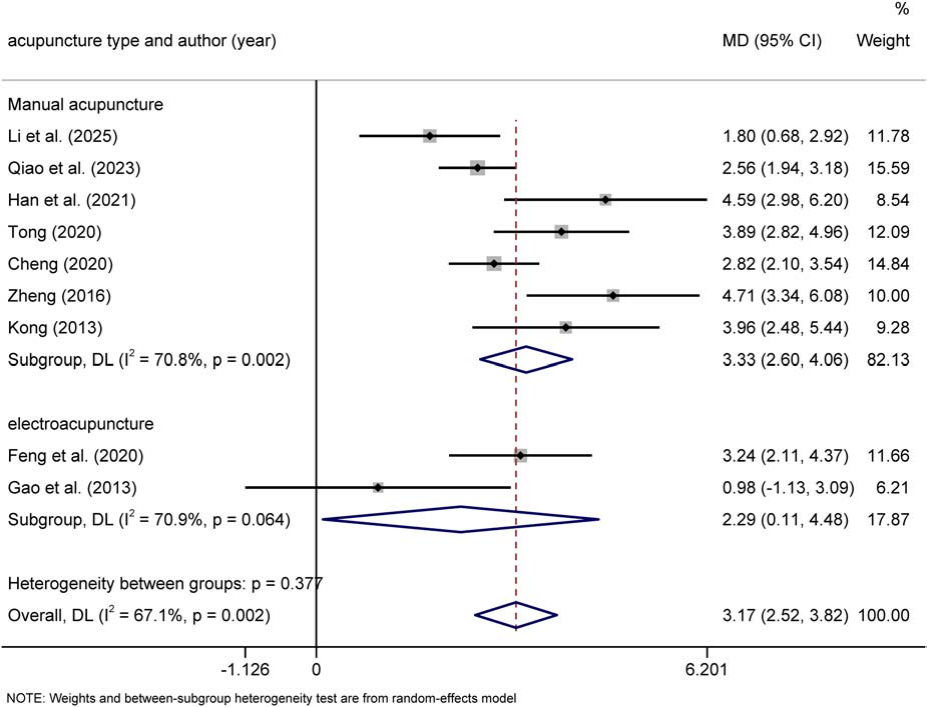
Forest plot of subgroup analysis of MMSE scores by acupuncture type in patients with PSVD.

**FIGURE 8:**
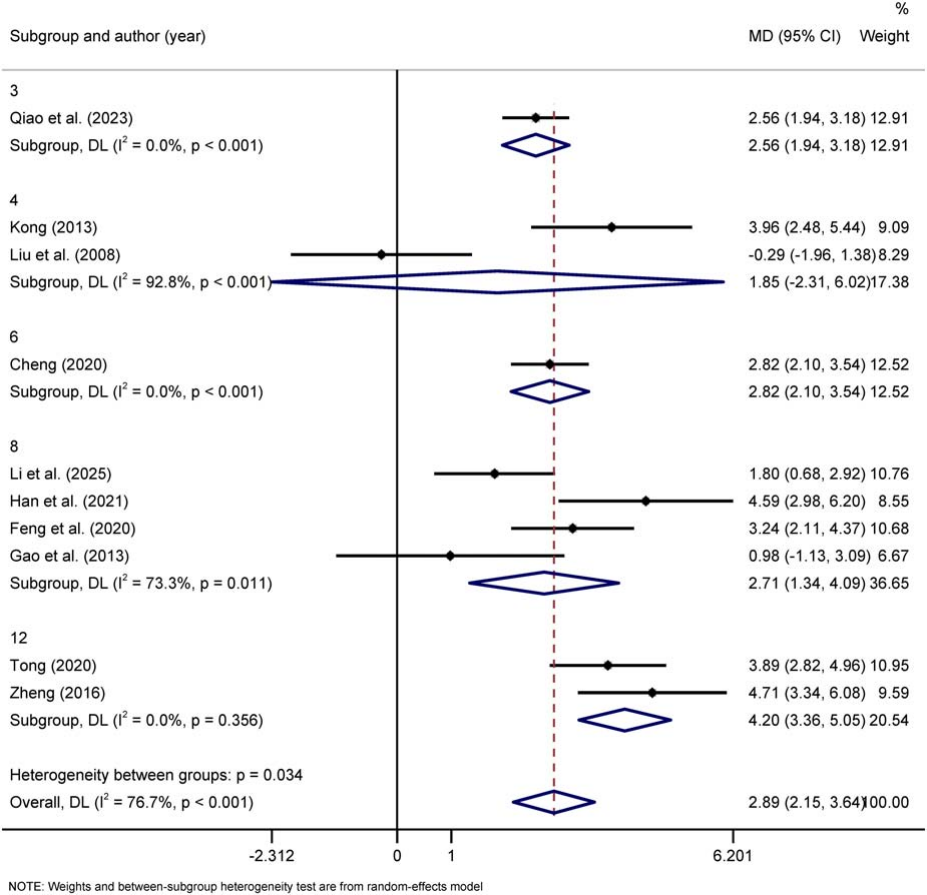
Forest plot of subgroup analysis of MMSE scores by treatment duration in patients with PSVD.

Ten studies involving 870 participants reported MMSE data (Liu et al., 2008; Gao et al., 2013; Kong, 2013; Zheng, 2016; Cheng, 2020; Feng and Wang, 2020; Tong, 2020; Han et al., 2021; Qiao and Hu, 2023; Li et al., 2025). The analytical outcomes are indicated in Figures 6–8. MMSE scores in the acupuncture group improved significantly compared with those in the control group (MD = 2.89; 95% CI = 2.15, 3.64; *p* < 0.001). Nevertheless, there was great heterogeneity (*I*^2^ = 76.7%). To further explore the source of heterogeneity, we conducted a subgroup analysis based on acupuncture type. Manual acupuncture showed a significant effect on improving MMSE scores (MD = 3.33, 95% CI: 2.60–4.06, *p* < 0.001, *I*^2^ = 70.8%), and electroacupuncture also yielded a significant effect (MD = 2.29, 95% CI: 0.11–4.48, *p* = 0.040, *I*^2^ = 70.9%). The between-subgroup heterogeneity test was not statistically significant (*p* = 0.377, *I*^2^ = 67.1%). A *post-hoc* exploratory subgroup analysis of treatment duration suggested a possible difference in effect size (*p* = 0.034). The 12-week subgroup showed a larger MMSE effect estimate (MD = 4.20, 95% CI = 3.36–5.05, *I*^2^ = 0%), whereas the 4-week subgroup showed no statistically significant effect (MD = 1.85, *p* = 0.383) and substantial heterogeneity (*I*^2^ = 92.8%). Because this subgroup analysis was *post-hoc* and not prespecified, these findings should be interpreted as hypothesis-generating rather than confirmatory.

#### 3.3.3 MoCA

**FIGURE 9:**
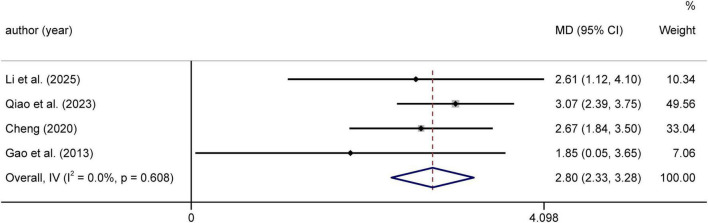
Forest plot of the effect of the experimental group and control group on MoCA of PSVD.

Four papers (Gao et al., 2013; Cheng, 2020; Qiao and Hu, 2023; Li et al., 2025) with 262 patients were considered in this study. These findings are illustrated in Figure 9. In the primary analysis, a fixed-effect model was used, and the robustness of this result was examined through fixed-effect versus random-effects sensitivity analyses. Compared with the control group, acupuncture therapy demonstrated significantly greater improvement in MoCA scores (MD = 2.80; 95% CI = 2.33, 3.28; *p* < 0.001). Since there was no heterogeneity among the selected studies, a subgroup analysis was unnecessary.

#### 3.3.4 TC, TG, and LDL-C

**FIGURE 10:**
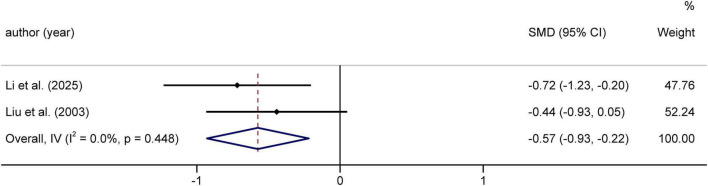
Forest plot of the effect of the experimental group and control group on TC of PSVD.

**FIGURE 11:**
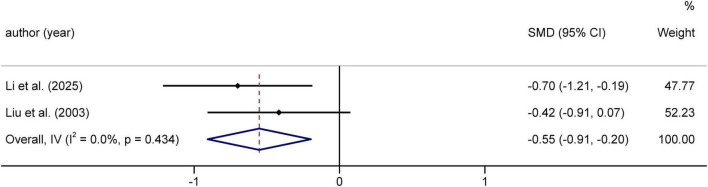
Forest plot of the effect of the experimental group and control group on TG of PSVD.

**FIGURE 12:**
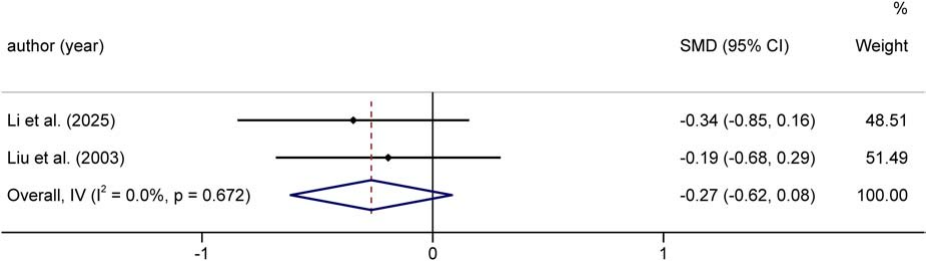
Forest plot of the effect of the experimental group and control group on LDL-C of PSVD.

Serum TC, TG, and LDL-C levels were analyzed in two studies (Liu, 2003; Li et al., 2025) of 130 patients with post-stroke dementia. The analytical results are presented in Figures 10– 12. In the primary analysis, fixed-effect models were used; these lipid-related estimates were further examined in fixed-effect versus random-effects sensitivity analyses and interpreted cautiously because they were based on only two studies. The analysis revealed that acupuncture significantly reduced total cholesterol (TC: SMD = −0.574, 95% CI = −0.929, −0.219, *p* = 0.002) and triglycerides (TG: SMD = −0.552, 95% CI = −0.907, −0.197, *p* = 0.002). However, low-density lipoprotein cholesterol (LDL-C) showed no significant treatment effect (SMD = −0.266, 95% CI = −0.616, 0.083, *p* = 0.135). All analyses showed complete homogeneity across studies (*I*^2^ = 0%; TC heterogeneity, *p* = 0.448; TG, *p* = 0.434; LDL-C, *p* = 0.672). These lipid-related findings may tentatively suggest a possible association between acupuncture and certain aspects of lipid metabolism; however, they should be regarded as exploratory because they are based on only two small studies with limited endpoints.

#### 3.3.5 Adverse events

Only three of the included studies reported adverse events (Liu et al., 2008; Feng and Wang, 2020; Li et al., 2025). Three cases of minor scalp hematoma occurred exclusively in the acupuncture intervention groups (see Supplementary Table 3 for details).

### 3.4 Sensitivity analysis

Sensitivity analyses included leave-one-out analyses and comparisons between fixed-effect and random-effects models. Leave-one-out plots for the study-defined overall effective rate, MMSE, MoCA, TC, and TG are presented in Supplementary Figures 1–5. Sensitivity analyses comparing fixed-effect and random-effects models showed that the direction and overall interpretation of the main findings were generally consistent across model choices, suggesting that the conclusions did not materially depend on the fixed-effect versus random-effects model selection. Lipid-related outcomes were still interpreted cautiously because they were based on only two studies (Supplementary Table 5).

### 3.5 Publication bias

**FIGURE 13:**
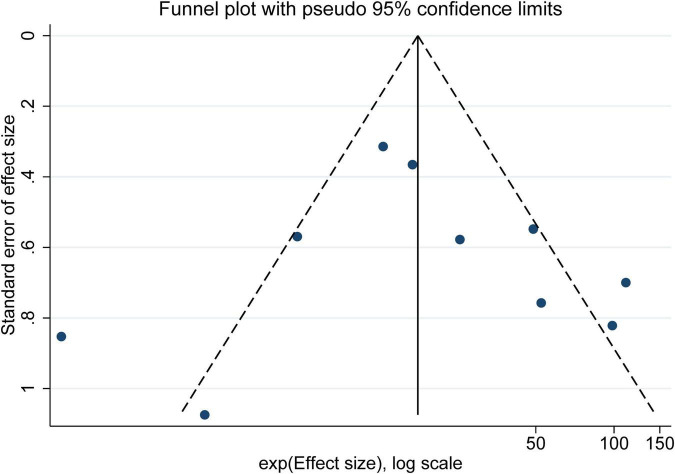
Funnel plot of MMSE scores in PSVD.

Publication bias was assessed only for the MMSE outcome, as it was the only analysis that included at least 10 studies. Visual inspection of the funnel plot suggested approximate symmetry (Figure 13). Begg’s test showed no evidence of publication bias (adjusted Kendall’s score = 1, z = 0.09, *p* = 0.929), and Egger’s test was also non-significant (bias coefficient = 0.181, 95% CI: −4.204 to 4.565, t = 0.09, *p* = 0.927). These findings did not suggest a strong signal of publication bias for MMSE, although residual bias cannot be excluded.

## 4 Discussion

This is the first systematic review and meta-analysis specifically evaluating acupuncture for post-stroke vascular dementia (PSVD), an underexplored area in dementia research. The present meta-analysis found that acupuncture-related interventions may be linked to better cognitive outcomes in patients with PSVD. Nevertheless, the results should be interpreted with caution, given the methodological limitations of the included trials, their regional concentration, incomplete adverse-event reporting, and the exploratory nature of the lipid-related and treatment-duration analyses.

The pooled analyses suggested that acupuncture interventions may be associated with improvements in several outcomes in patients with PSVD. Across trials evaluating acupuncture either as an add-on to conventional therapy or as a monotherapy comparator, acupuncture interventions were associated with a higher study-defined overall effective rate and improvements in both MMSE and MoCA scores. These two intervention designs address different clinical questions: add-on trials evaluate the incremental benefit of acupuncture beyond conventional therapy, whereas monotherapy trials evaluate acupuncture as the principal intervention compared with a non-acupuncture control. Therefore, pooled estimates combining these designs should be interpreted cautiously. In addition, the overall effective rate was a non-standardized, study-defined composite outcome based on heterogeneous response criteria across trials and should therefore be interpreted as exploratory and supportive rather than as a validated clinical endpoint. Acupuncture was also associated with lower total cholesterol and triglyceride levels, but these lipid-related findings were based on only two small studies and should be regarded as preliminary. Reported adverse events were few, but the safety findings should be interpreted cautiously given the incomplete reporting of adverse events across the included trials.

There was considerable heterogeneity, especially in the MMSE results (*I*^2^ = 76.7%). Subgroup analysis indicated that acupuncture type may not have been the main source of heterogeneity (*p* = 0.377). In a *post-hoc* exploratory subgroup analysis, treatment duration appeared to account for part of the observed heterogeneity, suggesting that treatment duration may be one factor associated with differences in cognitive benefit across studies. The 12-week subgroup showed a larger MMSE effect estimate (MD = 4.20, *I*^2^ = 0%), whereas the 4-week subgroup showed no statistically significant effect and substantial heterogeneity (*I*^2^ = 92.8%). However, because the treatment-duration analysis was exploratory and not prespecified, these findings should be interpreted as hypothesis-generating rather than confirmatory. Taken together, the subgroup findings raise the possibility that treatment duration may have contributed to differences in the magnitude and consistency of cognitive improvement across studies, although this interpretation requires confirmation in future trials that directly compare different treatment durations. In contrast, heterogeneity was low for other outcomes, including MoCA scores and lipid-related measures such as TC and TG.

Previous experimental studies have proposed several biological mechanisms that may be relevant to the effects of acupuncture in vascular cognitive impairment, including oxidative stress, neuroinflammation, synaptic plasticity, and vascular repair (He et al., 2017; Su et al., 2018; Yang et al., 2018; Gao et al., 2020; Bu et al., 2021; Custodero et al., 2022). These mechanisms may provide a possible context for interpreting the present findings, but they were not directly examined in the included studies and therefore remain speculative. The lipid-related findings should be regarded as exploratory because they were based on only two small studies with limited endpoints, and the larger MMSE effect observed in the 12-week subgroup should not be interpreted as definitive evidence of a duration-response relationship. Future studies with dedicated mechanistic endpoints are needed to clarify these issues.

Based on the limited adverse-event data reported in the included studies, acupuncture appeared to have an acceptable safety profile; however, this finding should be interpreted cautiously because adverse events were incompletely reported in most trials. Only three of the included studies reported adverse events. Three cases of minor scalp hematoma occurred exclusively in the acupuncture groups and resolved with simple local management. No serious acupuncture-related complications were reported. Nevertheless, the true incidence of adverse events may have been underestimated, as broader acupuncture safety studies have reported higher event rates than those captured in the present review.

The present review offers cautious, preliminary support for acupuncture as either an adjunctive option or a comparator intervention in PSVD. Nevertheless, the evidence base is not sufficiently robust to support firm conclusions. In the GRADE-informed appraisal, confidence in the findings was reduced by several factors, including methodological limitations in the included trials, clinical and methodological heterogeneity, imprecision due to the small number of studies for several outcomes, non-standardized definitions of the study-defined overall effective rate, and incomplete adverse-event reporting. First, the RoB 2 assessment further indicated that none of the included trials was judged to have an overall low risk of bias. Most studies had some concerns in the randomization process, which could lead to selection bias and inflated effect estimates, whereas 20% of the studies were judged to have a high risk of bias because of missing outcome data, raising concerns about attrition bias. Second, the study-defined overall effective rate was a non-standardized exploratory outcome based on heterogeneous response categories and calculation criteria across the nine included studies, as summarized in Supplementary Table 2. Although the statistical heterogeneity of this outcome was low, the interpretability of the pooled result is limited by the lack of a unified and validated endpoint definition, which may have introduced reporting and classification bias. Third, the subgroup analysis of treatment duration was a *post-hoc* exploratory analysis and was not prespecified, which increases the risk of false-positive findings due to multiple comparisons. Fourth, the assessment of publication bias for the MMSE outcome was limited by the small number of included studies (*k* = 10), which reduced the statistical power of the Egger and Begg tests. Although no strong signal of publication bias was detected, residual publication bias or small-study effects cannot be excluded. Fifth, the safety assessment is severely limited by incomplete adverse-event reporting in the primary trials. This likely led to under-ascertainment of adverse events, as broader acupuncture safety studies have reported higher incidence rates (8.6–9.31%) (Witt et al., 2009; Bäumler et al., 2021). Thus, our safety findings may underestimate the true risk of adverse events. Sixth, the lipid metabolism analysis is exploratory and based on only two included studies, which limits statistical power and generalizability. Seventh, the review lacked an in-depth analysis of differences among acupoint selection schemes and alternative interventions, including donepezil and nimodipine. In addition, because baseline data were insufficient, it was not possible to examine in detail whether the effects of acupuncture differed by stroke subtype or age group. Eighth, all included studies were conducted in China, which may limit the generalizability of our findings to other populations and settings.

Further progress in this field will depend on stronger clinical evidence. Large, multicenter randomized controlled trials with more rigorous design are needed to clarify the role of acupuncture in PSVD. In future studies, greater methodological rigor should include robust randomization procedures and, where feasible, the use of validated sham controls with appropriate blinding. Beyond trial quality itself, comparative effectiveness remains insufficiently understood. Direct comparisons between acupuncture and conventional pharmacotherapies are needed, and the potential value of combining acupuncture with drug treatment also warrants further study. Better patient stratification will be equally important. Future trials should prespecify subgroup analyses according to stroke subtype, including hemorrhagic and ischemic stroke, and should also investigate whether treatment responses differ by age or sex. Mechanistic studies are also needed. In particular, biomarker-based designs linking longitudinal changes in oxidative stress and inflammatory markers with cognitive outcomes may help clarify the biological basis of treatment response and whether treatment duration is related to outcome magnitude. In addition, long-term safety should be assessed using standardized adverse-event reporting criteria. Health economic studies may further help determine whether acupuncture can be integrated into clinical practice in a cost-effective way.

## 5 Conclusion

This study is the first meta-analysis specifically focused on acupuncture for PSVD. The available evidence points to possible benefits for cognitive outcomes, but the findings remain constrained by methodological weaknesses in the included studies, incomplete reporting of adverse events, and the exploratory nature of the lipid-related and treatment-duration results. Further large, high-quality, multicenter randomized controlled trials are needed to confirm these results and to clarify the role of acupuncture in the management of PSVD.

## Data Availability

The original contributions presented in this study are included in this article/Supplementary material, further inquiries can be directed to the corresponding author.
