# Supplementary material for: Effects of acupuncture on cognitive function and lipid metabolism in post-stroke vascular dementia: a systematic review and meta-analysis of randomized controlled trials
Source: Front Aging Neurosci. 2026 Jun 17;18:1797567. doi: 10.3389/fnagi.2026.1797567 (PMC13318961; doi:10.3389/fnagi.2026.1797567)
Supplement: Supplementary file 1 [file Data_Sheet_1.docx]

| Database | Table S1. Search Strategies for Each Database |
| --- | --- |
| CNKI | SU%=(‘针灸’ + ‘针’ + ‘针刺’ + ‘温针’ + ‘头针’ + ‘耳针’ + ‘电针’ + ‘穴位电刺激’ + ‘埋线’ + ‘耳穴’ + ‘体针’ + ‘腹针’ + ‘火针’ + ‘浮针’ + ‘刺络’）*（‘中风’ + ‘脑梗死’ + ‘脑缺血’ + ‘卒中’ + ‘脑出血’ + ‘脑血管障碍’ + ‘脑栓塞’ + ‘脑血栓’)*(‘痴呆’ + ‘血管性痴呆’) |
| Wanfang | 主题:(((“针灸” or “针” or “针刺” or “温针” or “头针” or “耳针” or “电针” or “穴位电刺激” or “埋线” or “耳穴” or “体针” or “腹针” or “火针” or “浮针” or “刺络”) and (“中风” or “脑梗死” or “脑缺血” or “卒中” or “脑出血” or “脑血管障碍” or “脑栓塞” or “脑血栓”)) and (“痴呆” or “血管性痴呆”) |
| VIP | M=(((“针灸” or “针” or “针刺” or “温针” or “头针” or “耳针” or “电针” or “穴位电刺激” or “埋线” or “耳穴” or “体针” or “腹针” or “火针” or “浮针” or “刺络”) and (“中风” or “脑梗死” or “脑缺血” or “卒中” or “脑出血” or “脑血管障碍” or “脑栓塞” or “脑血栓”)) and (“痴呆” or “血管性痴呆”)) |
| PubMed | (("Acupuncture Therapy"[Mesh]) OR (Acupuncture Therapy[Title/Abstract]) OR (Acupuncture[Title/Abstract]) OR (Pharmacopuncture[Title/Abstract]) OR (Acupuncture Treatment[Title/Abstract]) OR (Acupuncture Treatments[Title/Abstract]) OR (Treatment, Acupuncture[Title/Abstract]) OR (Therapy, Acupuncture[Title/Abstract]) OR (Pharmacoacupuncture Treatment[Title/Abstract]) OR (Treatment, Pharmacoacupuncture[Title/Abstract]) OR (Pharmacoacupuncture Therapy[Title/Abstract]) OR (Therapy, Pharmacoacupuncture[Title/Abstract]) OR (Acupotomy[Title/Abstract]) OR (Acupotomies[Title/Abstract]) OR (Acupunctures, Ear[Title/Abstract]) OR (Ear Acupunctures[Title/Abstract]) OR (Acupuncture, Auricular[Title/Abstract]) OR (Acupunctures, Auricular[Title/Abstract]) OR (Auricular Acupunctures[Title/Abstract]) OR (Auricular Acupuncture[Title/Abstract]) OR (Ear Acupuncture[Title/Abstract]) OR (Acupuncture Point[Title/Abstract]) OR (Point, Acupuncture[Title/Abstract]) OR (Points, Acupuncture[Title/Abstract]) OR (Acupoints[Title/Abstract]) OR (Acupoint[Title/Abstract]) OR (electroacupuncture[Title/Abstract]) OR (Moxibustion[Title/Abstract]) OR (warm acupuncture[Title/Abstract]) OR (Catgut Embedding[Title/Abstract]) OR (acupoint catgut embedding[Title/Abstract]) OR (press needling[Title/Abstract]) OR (manual acupuncture[Title/Abstract]) OR (wheat-grain moxibustion[Title/Abstract]) OR (indirect moxibustion[Title/Abstract]) OR (filiform needle[Title/Abstract]) OR (needle warming therapy[Title/Abstract]) OR (warm needle[Title/Abstract]) OR (floating acupuncture[Title/Abstract]) OR (Acupoint thread-embedding[Title/Abstract]) OR (Scalp acupuncture[Title/Abstract])) AND (("Stroke"[Mesh]) OR Stroke[Title/Abstract]) OR (Strokes[Title/Abstract]) OR (Cerebrovascular Accident[Title/Abstract]) OR (Cerebrovascular Accidents[Title/Abstract]) OR (Cerebral Stroke[Title/Abstract]) OR (Cerebral Strokes[Title/Abstract]) OR (Stroke, Cerebral[Title/Abstract]) OR (Strokes, Cerebral[Title/Abstract]) OR (Cerebrovascular Apoplexy[Title/Abstract]) OR (Apoplexy, Cerebrovascular[Title/Abstract]) OR (Vascular Accident, Brain[Title/Abstract]) OR (Brain Vascular Accident[Title/Abstract]) OR (Brain Vascular Accidents[Title/Abstract]) OR (Vascular Accidents, Brain[Title/Abstract]) OR (Cerebrovascular Stroke[Title/Abstract]) OR (Cerebrovascular Strokes[Title/Abstract]) OR (Stroke, Cerebrovascular[Title/Abstract]) OR (Strokes, Cerebrovascular[Title/Abstract]) OR (Apoplexy[Title/Abstract]) OR (CVA[Title/Abstract]) OR (CVAs[Title/Abstract]) OR (Stroke, Acute[Title/Abstract]) OR (Acute Stroke[Title/Abstract]) OR (Acute Strokes[Title/Abstract]) OR (Strokes, Acute[Title/Abstract]) OR (Cerebrovascular Accident, Acute[Title/Abstract]) OR (Acute Cerebrovascular Accident[Title/Abstract]) OR (Acute Cerebrovascular Accidents[Title/Abstract]) OR (Cerebrovascular Accidents, Acute[Title/Abstract])) AND (("Dementia, Vascular"[Mesh]) OR (Dementia, Vascular[Title/Abstract]) OR (Dementias, Vascular[Title/Abstract]) OR (Vascular Dementias[Title/Abstract]) OR (Vascular Dementia[Title/Abstract]) OR (Arteriosclerotic Dementia[Title/Abstract]) OR (Arteriosclerotic Dementias[Title/Abstract]) OR (Dementia, Arteriosclerotic[Title/Abstract]) OR (Dementias, Arteriosclerotic[Title/Abstract]) OR (Binswanger Disease[Title/Abstract]) OR (Disease, Binswanger[Title/Abstract]) OR (Encephalopathy, Binswanger[Title/Abstract]) OR (Chronic Progressive Subcortical Encephalopathy[Title/Abstract]) OR (Binswanger Encephalopathy[Title/Abstract]) OR (Leukoencephalopathy, Subcortical[Title/Abstract]) OR (Leukoencephalopathies, Subcortical[Title/Abstract]) OR (Subcortical Leukoencephalopathies[Title/Abstract]) OR (Encephalopathy, Subcortical Arteriosclerotic[Title/Abstract]) OR (Encephalopathy, Chronic Progressive Subcortical[Title/Abstract]) OR (Encephalopathy, Subcortical, Chronic Progressive[Title/Abstract]) OR (Subcortical Encephalopathy, Chronic Progressive[Title/Abstract]) OR (Subcortical Leukoencephalopathy[Title/Abstract]) OR (Subcortical Arteriosclerotic Encephalopathy[Title/Abstract]) OR (Arteriosclerotic Encephalopathy, Subcortical[Title/Abstract]) OR (Arteriosclerotic Encephalopathies, Subcortical[Title/Abstract]) OR (Encephalopathies, Subcortical Arteriosclerotic[Title/Abstract]) OR (Subcortical Arteriosclerotic Encephalopathies[Title/Abstract]) OR (Encephalopathy, Binswanger's[Title/Abstract]) OR (Binswanger's Encephalopathy[Title/Abstract]) OR (Encephalopathy, Binswangers[Title/Abstract]) OR (Binswanger's Disease[Title/Abstract]) OR (Binswangers Disease[Title/Abstract]) OR (Disease, Binswanger's[Title/Abstract]) OR (Subcortical Vascular Dementia[Title/Abstract]) OR (Dementias, Subcortical Vascular[Title/Abstract]) OR (Dementia, Subcortical Vascular[Title/Abstract]) OR (Subcortical Vascular Dementias[Title/Abstract]) OR (Vascular Dementias, Subcortical[Title/Abstract]) OR (Vascular Dementia, Subcortical[Title/Abstract]) OR (Vascular Dementia, Acute Onset[Title/Abstract]) OR (Acute Onset Vascular Dementia[Title/Abstract])) |
| Embase | ('Stroke':ti,ab,kw OR 'Strokes':ti,ab,kw OR 'Cerebrovascular Accident':ti,ab,kw OR 'Cerebrovascular Accidents':ti,ab,kw OR 'Cerebral Stroke':ti,ab,kw OR 'Cerebral Strokes':ti,ab,kw OR 'Stroke, Cerebral':ti,ab,kw OR 'Strokes, Cerebral':ti,ab,kw OR 'Cerebrovascular Apoplexy':ti,ab,kw OR 'Apoplexy, Cerebrovascular':ti,ab,kw OR 'Vascular Accident, Brain':ti,ab,kw OR 'Brain Vascular Accident':ti,ab,kw OR 'Brain Vascular Accidents':ti,ab,kw OR 'Vascular Accidents, Brain':ti,ab,kw OR 'Cerebrovascular Stroke':ti,ab,kw OR 'Cerebrovascular Strokes':ti,ab,kw OR 'Stroke, Cerebrovascular':ti,ab,kw OR 'Strokes, Cerebrovascular':ti,ab,kw OR 'Apoplexy':ti,ab,kw OR 'CVA':ti,ab,kw OR 'CVAs':ti,ab,kw OR 'Stroke, Acute':ti,ab,kw OR 'Acute Stroke':ti,ab,kw OR 'Acute Strokes':ti,ab,kw OR 'Strokes, Acute':ti,ab,kw OR 'Cerebrovascular Accident, Acute':ti,ab,kw OR 'Acute Cerebrovascular Accident':ti,ab,kw OR 'Acute Cerebrovascular Accidents':ti,ab,kw OR 'Cerebrovascular Accidents, Acute':ti,ab,kw) AND ('Acupuncture Therapy':ti,ab,kw OR 'Acupuncture':ti,ab,kw OR 'Pharmacopuncture':ti,ab,kw OR 'Acupuncture Treatment':ti,ab,kw OR 'Acupuncture Treatments':ti,ab,kw OR 'Treatment, Acupuncture':ti,ab,kw OR 'Therapy, Acupuncture':ti,ab,kw OR 'Pharmacoacupuncture Treatment':ti,ab,kw OR 'Treatment, Pharmacoacupuncture':ti,ab,kw OR 'Pharmacoacupuncture Therapy':ti,ab,kw OR 'Therapy, Pharmacoacupuncture':ti,ab,kw OR 'Acupotomy':ti,ab,kw OR 'Acupotomies':ti,ab,kw OR 'Acupunctures, Ear':ti,ab,kw OR 'Ear Acupunctures':ti,ab,kw OR 'Acupuncture, Auricular':ti,ab,kw OR 'Acupunctures, Auricular':ti,ab,kw OR 'Auricular Acupunctures':ti,ab,kw OR 'Auricular Acupuncture':ti,ab,kw OR 'Ear Acupuncture':ti,ab,kw OR 'Acupuncture Point':ti,ab,kw OR 'Point, Acupuncture':ti,ab,kw OR 'Points, Acupuncture':ti,ab,kw OR 'Acupoints':ti,ab,kw OR 'Acupoint':ti,ab,kw OR 'electroacupuncture':ti,ab,kw OR 'Moxibustion':ti,ab,kw OR 'warm acupuncture':ti,ab,kw OR 'Catgut Embedding':ti,ab,kw OR 'acupoint catgut embedding':ti,ab,kw OR 'press needling':ti,ab,kw OR 'manual acupuncture':ti,ab,kw OR 'wheat-grain moxibustion':ti,ab,kw OR 'indirect moxibustion':ti,ab,kw OR 'filiform needle':ti,ab,kw OR 'needle warming therapy':ti,ab,kw OR 'warm needle':ti,ab,kw OR 'floating acupuncture':ti,ab,kw OR 'Acupoint thread-embedding':ti,ab,kw OR 'Scalp acupuncture':ti,ab,kw) AND ('Dementia':ti,ab,kw OR 'Dementias':ti,ab,kw OR 'Amentia':ti,ab,kw OR 'Amentias':ti,ab,kw OR 'Senile Paranoid Dementia':ti,ab,kw OR 'Dementias, Senile Paranoid':ti,ab,kw OR 'Paranoid Dementia, Senile':ti,ab,kw OR 'Paranoid Dementias, Senile':ti,ab,kw OR 'Senile Paranoid Dementias':ti,ab,kw OR 'Familial Dementia':ti,ab,kw OR 'Dementia, Familial':ti,ab,kw OR 'Dementias, Familial':ti,ab,kw OR 'Familial Dementias':ti,ab,kw) |
| Cochrane Library | #1 (“Acupuncture Therapy” OR “Acupuncture” OR “Pharmacopuncture” OR “Acupuncture Treatment” OR “Acupuncture Treatments” OR “Treatment, Acupuncture” OR “Therapy, Acupuncture” OR “Pharmacoacupuncture Treatment” OR “Treatment, Pharmacoacupuncture” OR “Pharmacoacupuncture Therapy” OR “Therapy, Pharmacoacupuncture” OR “Acupotomy” OR “Acupotomies” OR “Acupunctures, Ear” OR “Ear Acupunctures” OR “Acupuncture, Auricular” OR “Acupunctures, Auricular” OR “Auricular Acupunctures” OR “Auricular Acupuncture” OR “Ear Acupuncture” OR “Acupuncture Point” OR “Point, Acupuncture” OR “Points, Acupuncture” OR “Acupoints” OR “Acupoint” OR “electroacupuncture” OR “Moxibustion” OR “warm acupuncture” OR “Catgut Embedding” OR “acupoint catgut embedding” OR “press needling” OR “manual acupuncture” OR “wheat-grain moxibustion” OR “indirect moxibustion” OR “filiform needle” OR “needle warming therapy” OR “warm needle” OR “floating acupuncture” OR “Acupoint thread-embedding” OR “Scalp acupuncture”):ti,ab,kw  #2 (“Stroke” OR “Strokes” OR “Cerebrovascular Accident” OR “Cerebrovascular Accidents” OR “Cerebral Stroke” OR “Cerebral Strokes” OR “Stroke, Cerebral” OR “Strokes, Cerebral” OR “Cerebrovascular Apoplexy” OR “Apoplexy, Cerebrovascular” OR “Vascular Accident, Brain” OR “Brain Vascular Accident” OR “Brain Vascular Accidents” OR “Vascular Accidents, Brain” OR “Cerebrovascular Stroke” OR “Cerebrovascular Strokes” OR “Stroke, Cerebrovascular” OR “Strokes, Cerebrovascular” OR “Apoplexy” OR “CVA” OR “CVAs” OR “Stroke, Acute” OR “Acute Stroke” OR “Acute Strokes” OR “Strokes, Acute” OR “Cerebrovascular Accident, Acute” OR “Acute Cerebrovascular Accident” OR “Acute Cerebrovascular Accidents” OR “Cerebrovascular Accidents, Acute”):ti,ab,kw  #3(“Dementia, Vascular” OR “Dementias, Vascular” OR “Vascular Dementias” OR “Vascular Dementia” OR “Arteriosclerotic Dementia” OR “Arteriosclerotic Dementias” OR “Dementia, Arteriosclerotic” OR “Dementias, Arteriosclerotic” OR “Binswanger Disease” OR “Disease, Binswanger” OR “Encephalopathy, Binswanger” OR “Chronic Progressive Subcortical Encephalopathy” OR “Binswanger Encephalopathy” OR “Leukoencephalopathy, Subcortical” OR “Leukoencephalopathies, Subcortical” OR “Subcortical Leukoencephalopathies” OR “Encephalopathy, Subcortical Arteriosclerotic” OR “Encephalopathy, Chronic Progressive Subcortical” OR “Encephalopathy, Subcortical, Chronic Progressive” OR “Subcortical Encephalopathy, Chronic Progressive” OR “Subcortical Leukoencephalopathy” OR “Subcortical Arteriosclerotic Encephalopathy” OR “Arteriosclerotic Encephalopathy, Subcortical” OR “Arteriosclerotic Encephalopathies, Subcortical” OR “Encephalopathies, Subcortical Arteriosclerotic” OR “Subcortical Arteriosclerotic Encephalopathies” OR “Encephalopathy, Binswanger's” OR “Binswanger's Encephalopathy” OR “Encephalopathy, Binswangers” OR “Binswanger's Disease” OR “Binswangers Disease” OR “Disease, Binswanger's” OR “Subcortical Vascular Dementia” OR “Dementias, Subcortical Vascular” OR “Dementia, Subcortical Vascular” OR “Subcortical Vascular Dementias” OR “Vascular Dementias, Subcortical” OR “Vascular Dementia, Subcortical” OR “Vascular Dementia, Acute Onset” OR “Acute Onset Vascular Dementia”):ti,ab,kw  #1 and #2 and #3 |
| Web of Science | TS= ((‘Acupuncture Therapy’ OR ‘Acupuncture’ OR ‘Pharmacopuncture’ OR ‘Acupuncture Treatment’ OR ‘Acupuncture Treatments’ OR ‘Treatment, Acupuncture’ OR ‘Therapy, Acupuncture’ OR ‘Pharmacoacupuncture Treatment’ OR ‘Treatment, Pharmacoacupuncture’ OR ‘Pharmacoacupuncture Therapy’ OR ‘Therapy, Pharmacoacupuncture’ OR ‘Acupotomy’ OR ‘Acupotomies’ OR ‘Acupunctures, Ear’ OR ‘Ear Acupunctures’ OR ‘Acupuncture, Auricular’ OR ‘Acupunctures, Auricular’ OR ‘Auricular Acupunctures’ OR ‘Auricular Acupuncture’ OR ‘Ear Acupuncture’ OR ‘Acupuncture Point’ OR ‘Point, Acupuncture’ OR ‘Points, Acupuncture’ OR ‘Acupoints’ OR ‘Acupoint’ OR ‘electroacupuncture’ OR ‘Moxibustion’ OR ‘warm acupuncture’ OR ‘Catgut Embedding’ OR ‘acupoint catgut embedding’ OR ‘press needling’ OR ‘manual acupuncture’ OR ‘wheat-grain moxibustion’ OR ‘indirect moxibustion’ OR ‘filiform needle’ OR ‘needle warming therapy’ OR ‘warm needle’ OR ‘floating acupuncture’ OR ‘Acupoint thread-embedding’ OR ‘Scalp acupuncture’) AND (‘Stroke’ OR ‘Strokes’ OR ‘Cerebrovascular Accident’ OR ‘Cerebrovascular Accidents’ OR ‘Cerebral Stroke’ OR ‘Cerebral Strokes’ OR ‘Stroke, Cerebral’ OR ‘Strokes, Cerebral’ OR ‘Cerebrovascular Apoplexy’ OR ‘Apoplexy, Cerebrovascular’ OR ‘Vascular Accident, Brain’ OR ‘Brain Vascular Accident’ OR ‘Brain Vascular Accidents’ OR ‘Vascular Accidents, Brain’ OR ‘Cerebrovascular Stroke’ OR ‘Cerebrovascular Strokes’ OR ‘Stroke, Cerebrovascular’ OR ‘Strokes, Cerebrovascular’ OR ‘Apoplexy’ OR ‘CVA’ OR ‘CVAs’ OR ‘Stroke, Acute’ OR ‘Acute Stroke’ OR ‘Acute Strokes’ OR ‘Strokes, Acute’ OR ‘Cerebrovascular Accident, Acute’ OR ‘Acute Cerebrovascular Accident’ OR ‘Acute Cerebrovascular Accidents’ OR ‘Cerebrovascular Accidents, Acute’) AND (‘Dementia, Vascular’ OR ‘Dementias, Vascular’ OR ‘Vascular Dementias’ OR ‘Vascular Dementia’ OR ‘Arteriosclerotic Dementia’ OR ‘Arteriosclerotic Dementias’ OR ‘Dementia, Arteriosclerotic’ OR ‘Dementias, Arteriosclerotic’ OR ‘Binswanger Disease’ OR ‘Disease, Binswanger’ OR ‘Encephalopathy, Binswanger’ OR ‘Chronic Progressive Subcortical Encephalopathy’ OR ‘Binswanger Encephalopathy’ OR ‘Leukoencephalopathy, Subcortical’ OR ‘Leukoencephalopathies, Subcortical’ OR ‘Subcortical Leukoencephalopathies’ OR ‘Encephalopathy, Subcortical Arteriosclerotic’ OR ‘Encephalopathy, Chronic Progressive Subcortical’ OR ‘Encephalopathy, Subcortical, Chronic Progressive’ OR ‘Subcortical Encephalopathy, Chronic Progressive’ OR ‘Subcortical Leukoencephalopathy’ OR ‘Subcortical Arteriosclerotic Encephalopathy’ OR ‘Arteriosclerotic Encephalopathy, Subcortical’ OR ‘Arteriosclerotic Encephalopathies, Subcortical’ OR ‘Encephalopathies, Subcortical Arteriosclerotic’ OR ‘Subcortical Arteriosclerotic Encephalopathies’ OR ‘Encephalopathy, Binswanger's’ OR ‘Binswanger's Encephalopathy’ OR ‘Encephalopathy, Binswangers’ OR ‘Binswanger's Disease’ OR ‘Binswangers Disease’ OR ‘Disease, Binswanger's’ OR ‘Subcortical Vascular Dementia’ OR ‘Dementias, Subcortical Vascular’ OR ‘Dementia, Subcortical Vascular’ OR ‘Subcortical Vascular Dementias’ OR ‘Vascular Dementias, Subcortical’ OR ‘Vascular Dementia, Subcortical’ OR ‘Vascular Dementia, Acute Onset’ OR ‘Acute Onset Vascular Dementia’) ) |
|  |  |
|  |  |

The literature search for this meta-analysis was performed up to November 3, 2025.

CNKI: China National Knowledge Infrastructure; Embase: Excerpta Medica Database; VIP: VIP Database for Chinese Technical Periodicals.
