# Supplementary material for: Effects of acupuncture on cognitive function and lipid metabolism in post-stroke vascular dementia: a systematic review and meta-analysis of randomized controlled trials
Source: Front Aging Neurosci. 2026 Jun 17;18:1797567. doi: 10.3389/fnagi.2026.1797567 (PMC13318961; doi:10.3389/fnagi.2026.1797567)
Supplement: Supplementary file 2 [file Data_Sheet_2.docx]

**Supplementary Table S2. Study-level definitions and calculation criteria for the non-standardized outcome “overall effective rate”**

| **Study** | **Response categories used in the original trial** | **Definition / criteria reported by original study** | **Numerator used for overall effective rate** | **Denominator** |
| --- | --- | --- | --- | --- |
| Li et al. (2025) | Markedly effective; effective ; ineffective | Efficacy was based on MMSE score increase: score increase = [(post-treatment score − pre-treatment score) / pre-treatment score] × 100%. Markedly effective: MMSE increase ≥50%; effective: MMSE increase ≥25% and <50%; ineffective: MMSE increase <25%. | Markedly effective + effective | Total number of participants in each group |
| Qiao et al. (2023) | Markedly effective ; effective ; ineffective | Based on the Guiding Principles for Clinical Research of New Chinese Medicines. The original study explicitly defined total effective rate as (markedly effective + effective) / total number of participants × 100%. | Markedly effective + effective | Total number of participants in each group |
| Han et al. (2021) | Basic control; markedly effective; effective; ineffective | Based on the Guiding Principles for Clinical Research of New Chinese Medicines. Basic control: major symptoms basically recovered, consciousness clear, orientation intact, answers relevant, quick response, self-care, able to undertake general social activities. Markedly effective: major symptoms mostly recovered, orientation basically intact, answers basically relevant, general response, self-care. Effective: major symptoms reduced or partly disappeared, answers basically relevant, basic self-care, but slow response and residual intellectual/personality impairment. Ineffective: no improvement or aggravation. | Basic control + markedly effective + effective | Total number of participants in each group |
| Feng et al. (2020) | Markedly effective; effective; ineffective | Cognitive-function efficacy was evaluated after 8 weeks of treatment. The study compared markedly effective, effective, and ineffective categories for cognitive-function response. A separate TCM syndrome efficacy assessment was also reported, but the cognitive-function efficacy outcome was used for this table. | Markedly effective + effective | Total number of participants in each group |
| Zheng (2016) | basically cured; significant improvement; improvement; no change; deterioration | Based on the therapeutic-effect criteria from the Fourth National Cerebrovascular Disease Academic Conference. The original study classified clinical response into five categories: basically cured, significant improvement, improvement, no change/ineffective, and deterioration. | Basically cured/recovered + significant improvement + improvement | Total number of participants in each group |
| Kong (2013) | Clinical recovery; markedly effective; effective; ineffective | Based on the clinical efficacy criteria for dementia in the Guiding Principles for Clinical Research of New Chinese Medicines. Clinical recovery: major symptoms basically returned to normal, consciousness clear, orientation intact, answers relevant, quick response, self-care, able to perform general social activities. Markedly effective: major symptoms mostly returned to normal, orientation mostly intact, answers basically relevant, general response, self-care. Effective: major symptoms reduced or partly returned to normal, answers basically relevant, basic self-care, but slow response and residual intellectual/personality impairment. Ineffective: no improvement or aggravation. | Clinical recovery + markedly effective + effective | Total number of participants in each group |
| Liu et al. (2008) | Markedly effective; effective; ineffective; deterioration | Efficacy index was calculated by the nimodipine method: [(post-treatment score − pre-treatment score) / pre-treatment score] × 100%. Markedly effective: efficacy index ≥20%; effective: efficacy index ≥12%; ineffective: efficacy index <12%; deterioration category also reported. | Markedly effective + effective | Completed participants in each group |
| Lun et al. (2004a) | Markedly effective; effective; improved; ineffective | Based on criteria for diagnosis, syndrome differentiation, and curative-effect evaluation of senile dementia. Markedly effective: major symptoms basically recovered, consciousness clear, orientation intact, correct answers, flexible response, self-care, able to undertake general social activities. Effective: major mental symptoms reduced or partly disappeared, basic self-care, basically correct answers, but slow response and residual intellectual/personality impairment. Improved: major mental symptoms reduced, poor self-care ability, slow response, residual intellectual/personality impairment. Ineffective: no change or disease progression. | Markedly effective + effective + improved | Total number of participants in each group |
| Lun et al. (2004b) | Markedly effective; effective; ineffective | Based on the criteria for diagnosis, syndrome differentiation, and curative-effect evaluation of senile dementia. Markedly effective: major symptoms basically disappeared, consciousness clear, orientation intact, correct answers, flexible response, self-care, able to undertake general social activities. Effective: major mental symptoms reduced or partly disappeared, basic self-care, basically correct answers, but slow response and residual intellectual/personality impairment. Ineffective: no change or disease progression. | Markedly effective + effective | Total number of participants in each group |

Note. Overall effective rate was extracted according to the response categories and calculation criteria reported in each original trial. Because the categories and numerators varied across studies, this outcome was treated as a study-defined, non-standardized exploratory outcome rather than a validated clinical endpoint. Only trials reporting overall effective rate are listed in this table.
