# Supplementary material for: Effects of acupuncture on cognitive function and lipid metabolism in post-stroke vascular dementia: a systematic review and meta-analysis of randomized controlled trials
Source: Front Aging Neurosci. 2026 Jun 17;18:1797567. doi: 10.3389/fnagi.2026.1797567 (PMC13318961; doi:10.3389/fnagi.2026.1797567)
Supplement: Supplementary file 3 [file Data_Sheet_3.docx]

**Supplementary Table S3. Reported adverse events in the included studies.**

| **Study** | **Acupuncture group (events/total)** | **Control group (events/total)** | **Reported adverse events and management** |
| --- | --- | --- | --- |
| Li et al., 2025 | 0/31 | 0/31 | None reported. |
| Feng and Wang, 2020 | 3/30 | 0/30 | Three transient scalp hematomas in the acupuncture group; all resolved with simple cotton-ball compression and did not require additional treatment or discontinuation of therapy. |
| Liu et al., 2008 | 0/47 | Not reported | No adverse events were reported in the acupuncture group; control-group adverse-event data were not reported. |
| Remaining included studies | Not reported | Not reported | Adverse-event data were not reported. |
| **Summary** | **3/108** | **0/61** | **Summary based only on studies that explicitly reported adverse-event data.** |

**Note:** Reported adverse events were available in only a small number of included studies. These data likely underestimate the true incidence of adverse events because most trials did not provide systematic safety reporting.
