# Supplementary material for: Effects of acupuncture on cognitive function and lipid metabolism in post-stroke vascular dementia: a systematic review and meta-analysis of randomized controlled trials
Source: Front Aging Neurosci. 2026 Jun 17;18:1797567. doi: 10.3389/fnagi.2026.1797567 (PMC13318961; doi:10.3389/fnagi.2026.1797567)
Supplement: Supplementary file 4 [file Data_Sheet_4.docx]

**Supplementary Table S4. GRADE-informed narrative appraisal of the certainty of evidence for the main outcomes**

| **Outcome** | **Evidence base** | **Main pooled finding** | **Risk of bias** | **Inconsistency** | **Indirectness** | **Imprecision** | **Publication bias** | **Narrative certainty**  **judgement** | **Interpretation** |
| --- | --- | --- | --- | --- | --- | --- | --- | --- | --- |
| Study-defined overall  effective rate | 9 RCTs; 769 participants | RR = 1.27,  95% CI 1.17–1.37;  I² = 28.7% | Serious concern: no included trial was rated as overall low risk of bias; allocation concealment was insufficiently reported in most trials. | Not serious based on statistical heterogeneity, although clinical definitions varied. | Serious concern: this was a non-standardized, study-defined composite outcome with heterogeneous response criteria across trials. | Not serious: the CI did not cross the null effect and the sample size was moderate. | Undetected/unclear: fewer than 10 studies were included, so formal assessment was limited. | Low certainty; exploratory outcome | The pooled result suggests a higher study-defined response rate with acupuncture-related interventions, but this outcome should be interpreted as exploratory and supportive only, not as a standardized clinical endpoint. |
| MMSE | 10 RCTs; 870 participants | MD = 2.89,  95% CI 2.15–3.64;  I² = 76.7% | Serious concern: most studies had some concerns or high risk of bias, mainly related to randomization/allocation concealment and missing data. | Serious concern: substantial statistical heterogeneity was present. | Not serious for the target population and cognitive outcome, although all studies were conducted in China. | Not serious: the CI was relatively precise and did not cross the null effect. | No strong signal detected by funnel plot, Begg’s test, or Egger’s test, but residual bias cannot be excluded. | Low certainty | The evidence suggests a possible improvement in MMSE scores, but the result should be interpreted cautiously because of risk of bias and substantial heterogeneity. |
| MoCA | 4 RCTs; 262 participants | MD = 2.80,  95% CI 2.33–3.28;  I² = 0% | Serious concern: the same overall methodological limitations of the included trials apply. | Not serious based on statistical heterogeneity. | Not serious for the cognitive outcome. | Serious concern: the number of studies and participants was small. | Undetected/unclear: too few studies were available for formal publication-bias assessment. | Low certainty | The evidence suggests a possible improvement in MoCA scores, but the certainty remains limited because the estimate is based on a small evidence base. |
| Total cholesterol (TC) | 2 RCTs; 130 participants | SMD = −0.574,  95% CI −0.929 to −0.219;  I² = 0% | Serious concern: included studies were subject to methodological limitations. | Not serious based on statistical heterogeneity, but only two studies were available. | Serious concern: lipid metabolism was a secondary and mechanistically indirect endpoint for PSVD treatment efficacy. | Serious concern: very small number of studies and participants. | Undetected/unclear: too few studies were available for assessment. | Very low certainty; exploratory evidence | The finding may suggest a possible association between acupuncture and lower TC, but this should be regarded as preliminary and exploratory. |
| Triglycerides (TG) | 2 RCTs; 130 participants | SMD = −0.552,  95% CI −0.907 to −0.197;  I² = 0% | Serious concern: included studies were subject to methodological limitations. | Not serious based on statistical heterogeneity, but only two studies were available. | Serious concern: lipid metabolism was not a direct clinical cognitive endpoint. | Serious concern: very small evidence base. | Undetected/unclear: too few studies were available for assessment. | Very low certainty; exploratory evidence | The finding may suggest a possible association between acupuncture and lower TG, but it should not be considered confirmatory. |
| Low-density lipoprotein cholesterol  (LDL-C) | 2 RCTs; 130 participants | SMD = −0.266,  95% CI −0.616 to 0.083;  I² = 0% | Serious concern: included studies were subject to methodological limitations. | Not serious based on statistical heterogeneity, but only two studies were available. | Serious concern: lipid metabolism was not a direct clinical cognitive endpoint. | Serious concern: very small evidence base and CI included no statistically significant effect. | Undetected/unclear: too few studies were available for assessment. | Very low certainty; exploratory evidence | The available evidence is insufficient to determine whether acupuncture affects LDL-C levels. |
| Adverse events | 3 studies reported  adverse-event data | Three minor scalp hematomas were reported; no serious  acupuncture-related adverse events were reported. | Serious concern: adverse-event monitoring and reporting were incomplete across the included trials. | Not estimable: adverse events were not consistently reported. | Serious concern: lack of standardized safety monitoring limits direct safety inference. | Serious concern: very sparse event data and incomplete reporting. | Suspected/unclear: selective non-reporting of adverse events cannot be excluded. | Very low certainty | The available data are insufficient to draw firm conclusions about safety; the true incidence of adverse events may have been underestimated. |

Note. RCT, randomized controlled trial; RR, risk ratio; MD, mean difference; SMD, standardized mean difference; CI, confidence interval; MMSE, Mini-Mental State Examination; MoCA, Montreal Cognitive Assessment; TC, total cholesterol; TG, triglycerides; LDL-C, low-density lipoprotein cholesterol.
