# Supplementary material for: Effects of acupuncture on cognitive function and lipid metabolism in post-stroke vascular dementia: a systematic review and meta-analysis of randomized controlled trials
Source: Front Aging Neurosci. 2026 Jun 17;18:1797567. doi: 10.3389/fnagi.2026.1797567 (PMC13318961; doi:10.3389/fnagi.2026.1797567)
Supplement: Supplementary file 5 [file Data_Sheet_5.docx]

**Supplementary Table S5. Sensitivity analyses comparing fixed-effect and random-effects models for main outcomes**

| **Outcome** | **Effect measure** | **Primary model used in main analysis** | **Fixed-effect estimate (95% CI)** | **Random-effects estimate (95% CI)** | **Interpretation** |
| --- | --- | --- | --- | --- | --- |
| Study-defined overall effective rate | RR | Fixed-effect | 1.27 (1.17 to 1.37) | 1.25 (1.14 to 1.37) | Direction and statistical interpretation unchanged |
| MMSE | MD | Random-effects | 2.87 (2.53 to 3.20) | 2.89 (2.15 to 3.64) | Direction and statistical interpretation unchanged |
| MoCA | MD | Fixed-effect | 2.80 (2.33 to 3.28) | 2.80 (2.33 to 3.28) | Direction and statistical interpretation unchanged |
| Total cholesterol (TC) | SMD | Fixed-effect | -0.57 (-0.93 to -0.22) | -0.57 (-0.93 to -0.22) | Direction unchanged; exploratory lipid-related finding |
| Triglycerides (TG) | SMD | Fixed-effect | -0.55 (-0.91 to -0.20) | -0.55 (-0.91 to -0.20) | Direction unchanged; exploratory lipid-related finding |
| Low-density lipoprotein cholesterol (LDL-C) | SMD | Fixed-effect | -0.27 (-0.62 to 0.08) | -0.27 (-0.62 to 0.08) | Direction unchanged; remained statistically non-significant |

Note. Values are based on the combined estimates from sensitivity analyses. RR, risk ratio; MD, mean difference; SMD, standardized mean difference; CI, confidence interval; MMSE, Mini-Mental State Examination; MoCA, Montreal Cognitive Assessment; TC, total cholesterol; TG, triglycerides; LDL-C, low-density lipoprotein cholesterol. Lipid-related outcomes were based on only two studies and should be interpreted as exploratory.
