# Supplementary figures and images for: Effects of acupuncture on cognitive function and lipid metabolism in post-stroke vascular dementia: a systematic review and meta-analysis of randomized controlled trials
Source: Front Aging Neurosci. 2026 Jun 17;18:1797567. doi: 10.3389/fnagi.2026.1797567 (PMC13318961; doi:10.3389/fnagi.2026.1797567)

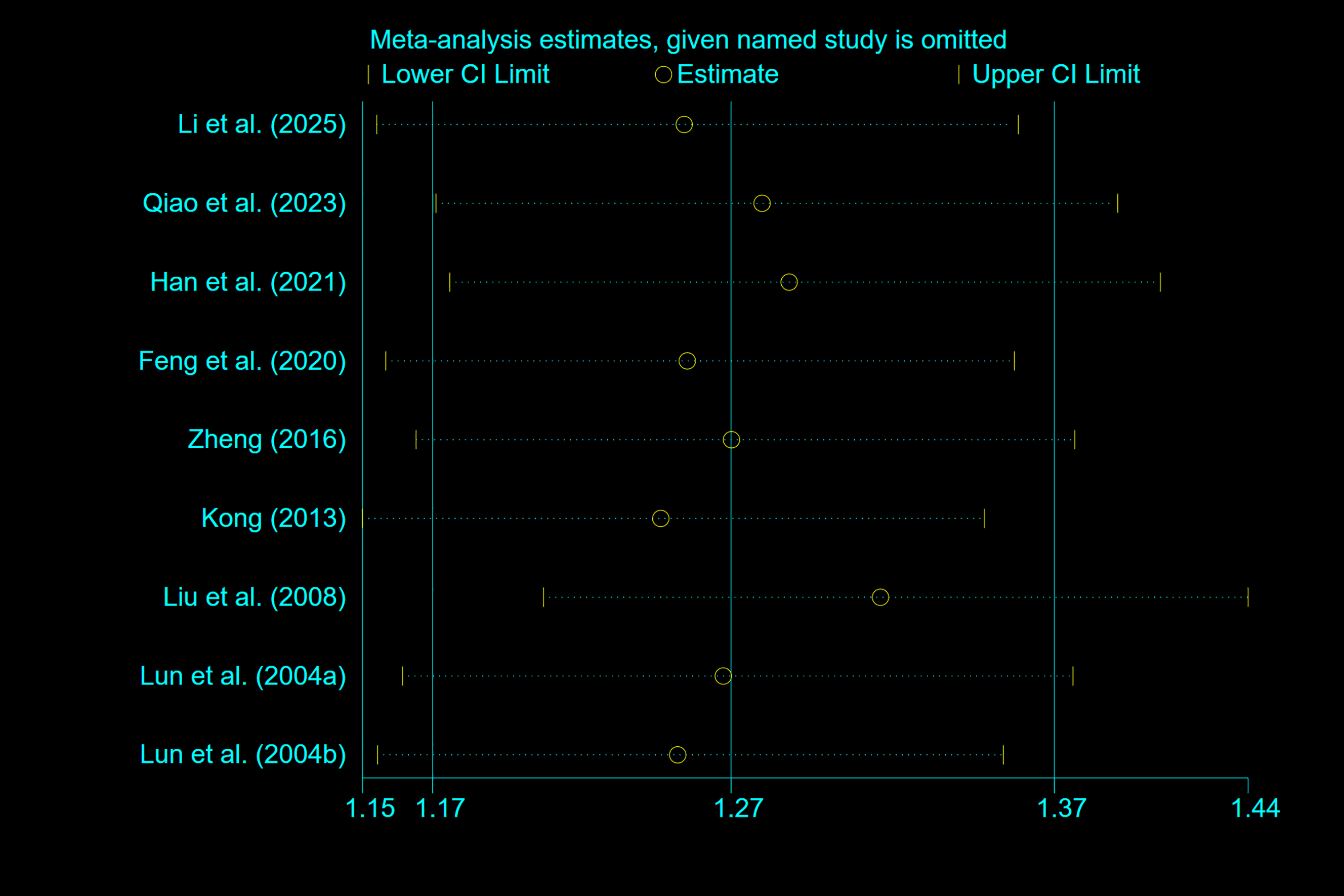

Supplement: Supplementary file 6 [file Image_1.tiff]

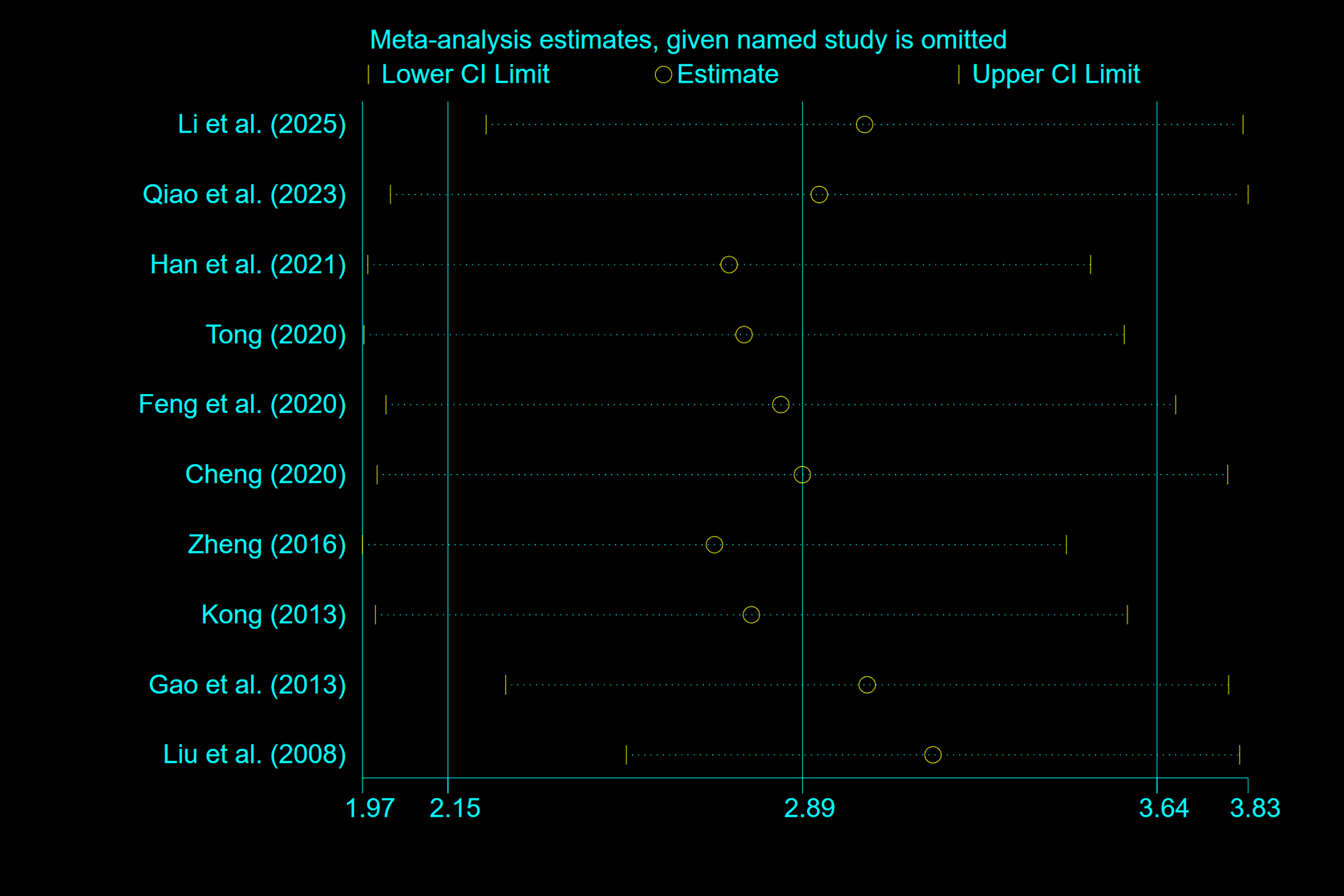

Supplement: Supplementary file 7 [file Image_2.tiff]

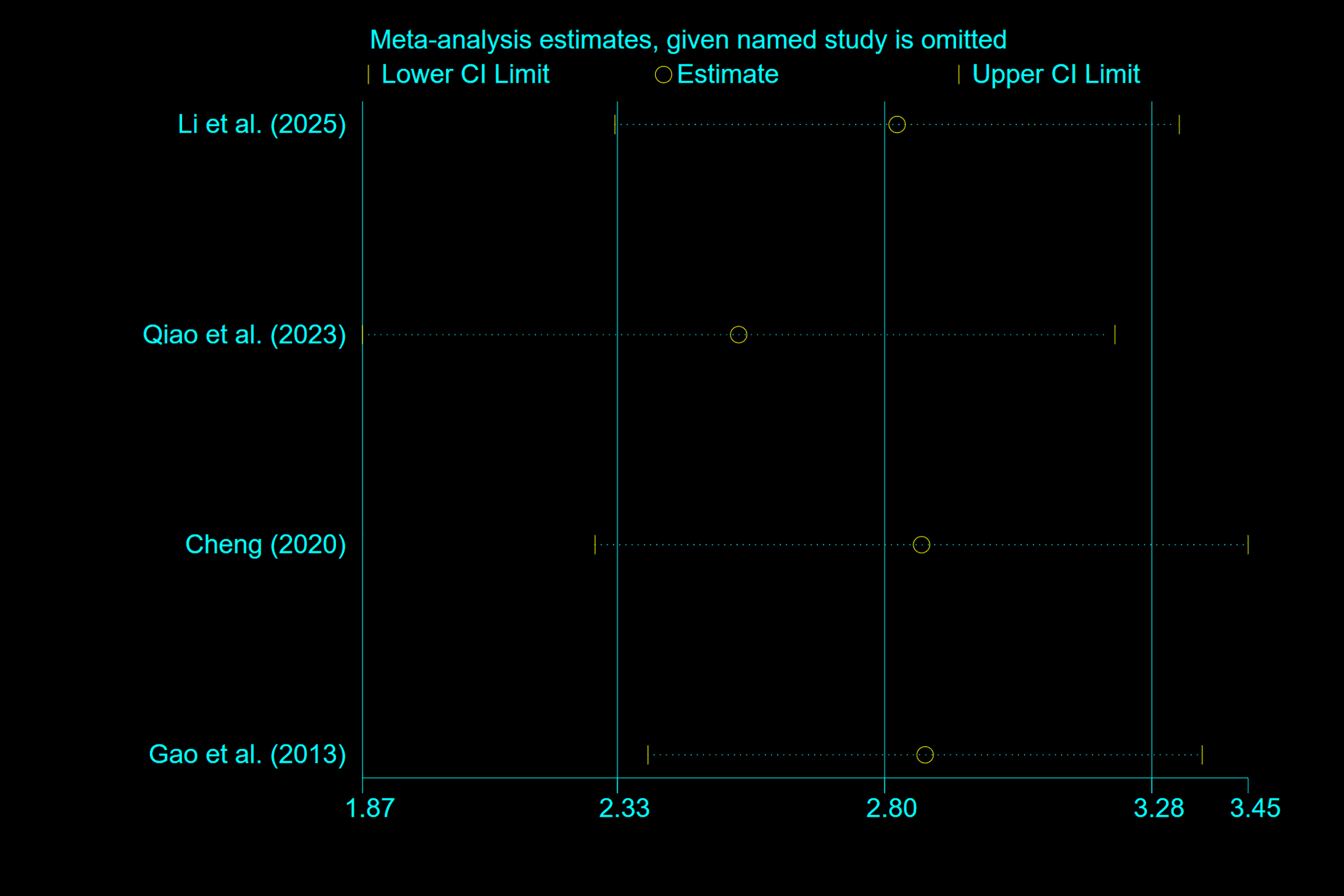

Supplement: Supplementary file 8 [file Image_3.tiff]

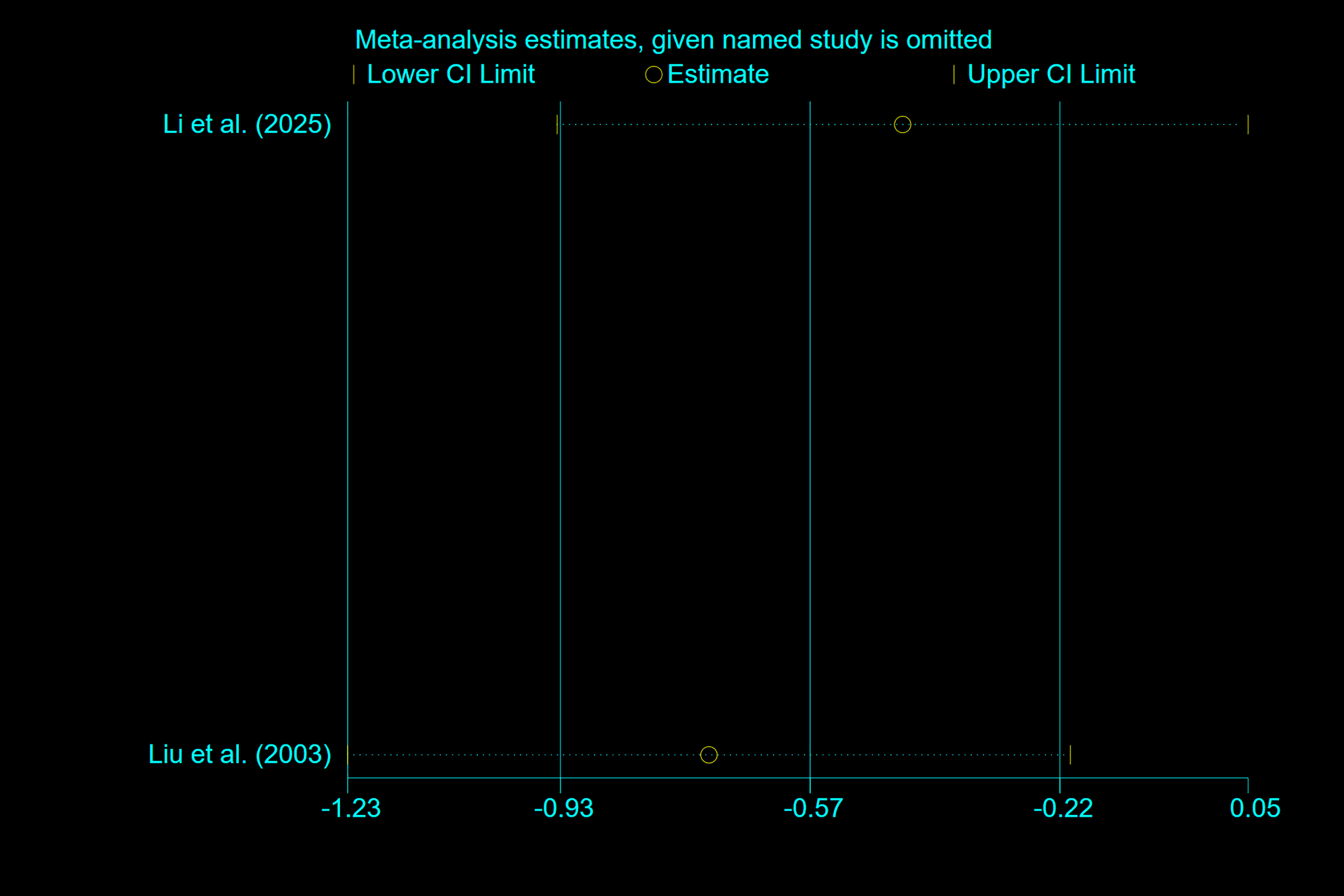

Supplement: Supplementary file 9 [file Image_4.tiff]

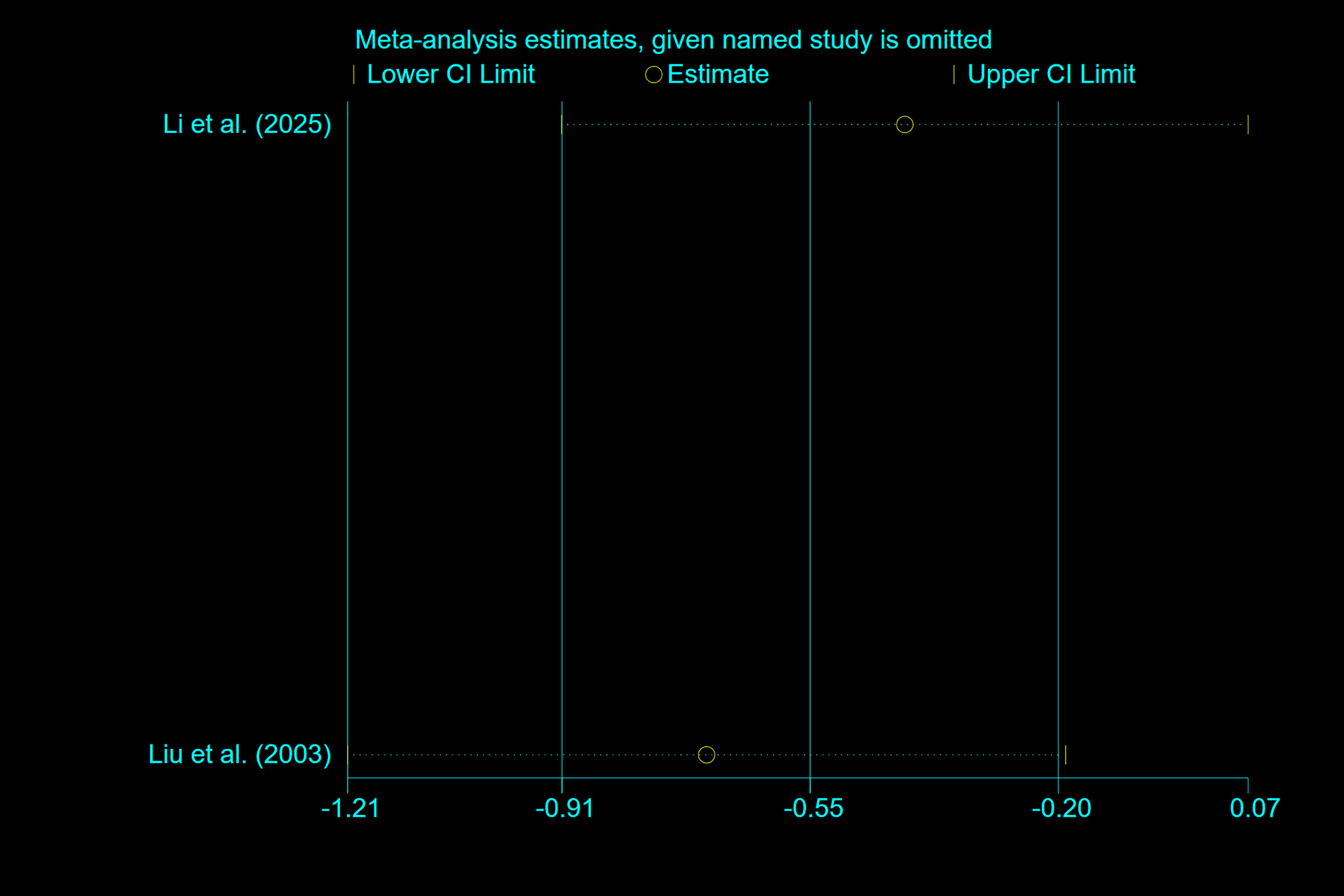

Supplement: Supplementary file 10 [file Image_5.tiff]
