# Supplementary material for: Effects of acupuncture on cognitive function and lipid metabolism in post-stroke vascular dementia: a systematic review and meta-analysis of randomized controlled trials
Source: Front Aging Neurosci. 2026 Jun 17;18:1797567. doi: 10.3389/fnagi.2026.1797567 (PMC13318961; doi:10.3389/fnagi.2026.1797567)
Supplement: Supplementary file 13 [file Supplementary_file_3.docx]

| **Abbreviation** | **Full Name** |
| --- | --- |
| ACh | Acetylcholine |
| Ang-1 | Angiopoietin-1 |
| CI | Confidence interval |
| DALYs | Disability-adjusted life years |
| DA | Dopamine |
| DSM-V | Diagnostic and Statistical Manual of Mental Disorders, Fifth Edition |
| fMRI | Functional magnetic resonance imaging |
| G6PD | Glucose-6-phosphate dehydrogenase |
| ICH | Intracerebral hemorrhage |
| ICD-11 | International Classification of Diseases, 11th Revision |
| IL-10 | Interleukin-10 |
| IL-1β | Interleukin-1β |
| LDL-C | Low-density lipoprotein cholesterol |
| LTP | Long-term potentiation |
| MD | Mean difference |
| M/F | Male/Female |
| MMSE | Mini-Mental State Examination |
| MoCA | Montreal Cognitive Assessment |
| NA | Not available |
| NADPH | Nicotinamide adenine dinucleotide phosphate |
| NMDA | N-methyl-D-aspartate |
| nACh | Nicotinic acetylcholine |
| NF-kappa B | Nuclear factor-kappa B |
| Nrf2/HO-1 | Nuclear factor erythroid 2-related factor 2/Heme oxygenase-1 |
| PI3K/AKT | Phosphatidylinositol 3-kinase/Protein kinase B |
| PRISMA | Preferred Reporting Items for Systematic Reviews and Meta-Analyses |
| PROSPERO | International Prospective Register of Systematic Reviews |
| RCTs | Randomized controlled trials |
| RoB 2 | Cochrane Risk of Bias Tool 2.0 |
| ROS | Reactive oxygen species |
| RR | Relative risk |
| PSVD | Post-stroke vascular dementia |
| SMD | Standard mean difference |
| TC | Total cholesterol |
| TG | Triglycerides |
| TLR4 | Toll-like receptor 4 |
| TNF-α | Tumor necrosis factor-alpha |
| Trx-1/TrxR-1 | Thioredoxin-1/Thioredoxin reductase-1 |
| TXNIP | Thioredoxin-interacting protein |
| VaD | Vascular dementia |
| VCI | Vascular cognitive impairment |
| VEGF | Vascular endothelial growth factor |
| WMD | Weighted mean difference |
| 5-HT | 5-Hydroxytryptamine |
